# A System-on-Chip Based Hybrid Neuromorphic Compute Node Architecture for Reproducible Hyper-Real-Time Simulations of Spiking Neural Networks

**DOI:** 10.3389/fninf.2022.884033

**Published:** 2022-06-29

**Authors:** Guido Trensch, Abigail Morrison

**Affiliations:** ^1^Simulation and Data Laboratory Neuroscience, Jülich Supercomputing Centre, Institute for Advanced Simulation, Jülich Research Centre, Jülich, Germany; ^2^Department of Computer Science 3—Software Engineering, RWTH Aachen University, Aachen, Germany; ^3^Institute of Neuroscience and Medicine (INM-6), Institute for Advanced Simulation (IAS-6), JARA-Institute Brain Structure-Function Relationship (JBI-1/INM-10), Research Centre Jülich, Jülich, Germany

**Keywords:** neuromorphic computing, compute node, FPGA, SoC, spiking neural networks, simulation, performance, parallel computing

## Abstract

Despite the great strides neuroscience has made in recent decades, the underlying principles of brain function remain largely unknown. Advancing the field strongly depends on the ability to study large-scale neural networks and perform complex simulations. In this context, simulations in hyper-real-time are of high interest, as they would enable both comprehensive parameter scans and the study of slow processes, such as learning and long-term memory. Not even the fastest supercomputer available today is able to meet the challenge of accurate and reproducible simulation with hyper-real acceleration. The development of novel neuromorphic computer architectures holds out promise, but the high costs and long development cycles for application-specific hardware solutions makes it difficult to keep pace with the rapid developments in neuroscience. However, advances in System-on-Chip (SoC) device technology and tools are now providing interesting new design possibilities for application-specific implementations. Here, we present a novel hybrid software-hardware architecture approach for a neuromorphic compute node intended to work in a multi-node cluster configuration. The node design builds on the Xilinx Zynq-7000 SoC device architecture that combines a powerful programmable logic gate array (FPGA) and a dual-core ARM Cortex-A9 processor extension on a single chip. Our proposed architecture makes use of both and takes advantage of their tight coupling. We show that available SoC device technology can be used to build smaller neuromorphic computing clusters that enable hyper-real-time simulation of networks consisting of tens of thousands of neurons, and are thus capable of meeting the high demands for modeling and simulation in neuroscience.

## 1. Introduction

In the process of gaining insight into the underlying principles of neural computation, the tools and methods developed and provided by computational neuroscience play a key role. In particular, we rely on the mathematical modeling of neuron, synapse, and neural network models and their numerical simulation to study their complex interaction and network dynamics. Community software for modeling, such as NeuroML (Gleeson et al., 2010), NMODL (Hines and Carnevale, 2000), and NESTML (Plotnikov et al., 2016), and for simulation, such as NEURON (Hines and Carnevale, 1997), Arbor (Akar et al., 2019), NEST (Gewaltig and Diesmann, 2007), and Brian (Goodman and Brette, 2008) provide such tools. They are complemented by numerical tools for statistical analysis, such as the Electrophysiology Analysis Toolkit *Elephant*^1^ as well as tool support for model validation methodologies, for example, the validation framework *NetworkUnit*^2^ (Gutzen et al., 2018). The requirements in regard to efficiency, correctness, and replicability and reproducibility of the outcomes place high demands on the whole software ecosystem.

When investigating large scale networks, in general one would like to simulate them as fast as possible. Whereas, real-time simulation is interesting because of the possibility of interacting with real-world applications, hyper-real-time would enable the study of slow processes, such as structural plasticity and long-term memory, and permit researchers to perform more comprehensive parameter scans of faster processes. This is still a major technical challenge (Friedmann et al., 2017), and not even the fastest supercomputer available today is up to the task.

Consequently, neuromorphic computing and application-specific novel hardware architectures are very attractive as they promise significant acceleration. However, the technical hurdles to making neuromorphic computing a useful tool for neuroscientists are not insignificant either. Crucially, flexibility and efficiency, which are both required for such a system, are opposing goals in the choice of technology (e.g., GPP^3^, FPGA^4^ or ASIC^5^; Noll et al., 2010). Optimal flexibility is achieved with traditional general purpose processors. The SpiNNaker system (Furber et al., 2013) is an example for a neuromorphic massively parallel computing platform that is based on digital multi-core chips using ARM processing cores. It is fully programmable, thus flexible in the choice and implementation of the numerical models, and allows large-scale simulations to be performed in real-time. The Heidelberg BrainScaleS system (Schemmel et al., 2010) and its successor BrainScales-2 (Pehle et al., 2022), in contrast, are capable of running simulations orders of magnitude faster than real-time. To achieve this, the architecture builds on the physical, i.e., analog, emulation of neuron and synapse models (Schemmel et al., 2017) in dedicated mixed-signal circuits combined with digital plasticity processors (BrainsScaleS-2) using a “hybrid plasticity” scheme (Friedmann et al., 2017). Physical, analog emulation thereby restricts the system to its built-in, “silicon-frozen” analog models, and use-cases where technology-related effects, such as fabrication tolerances and thermal noise, are acceptable.

During recent years, programmable device technology and tools have greatly increased in functionality, benefiting from the continued advances in semiconductor technology. Modern field programmable gate arrays (FPGAs) provide a large number of chip resources (e.g., logic cells and memories) allowing to implement complex hardware designs at affordable costs. High-level synthesis (HLS) tools allow the developer to generate hardware implementations from algorithmic descriptions, thus reducing development time and making the technology accessible to non hardware experts. Although the design effort remains high, programmable device technology offers a good compromise between flexibility and efficiency and has therefore been widely recognized as potentially well-suited to neural network simulation. This has been exploited by a number of digital neuromorphic architectures developed in recent years.

In an earlier study, Maguire et al. (2007) made an inventory and revealed the challenges associated with implementing large-scale spiking neural networks on FPGAs, emphasizing the importance of design decisions on system level and its impact on the final performance. Since then, a number of architectural approaches and implementations for different use cases have been published. A scalable modular architecture for closed-loop experiments with *in vitro* cultures is presented in Pani et al. (2017). The platform is able to simulate small-to-medium size networks in real-time, implementing 1,440 Izhikevich neurons. *Bluehive* (Moore et al., 2012)—a scalable custom 64-FPGA machine—is dedicated to the simulation of large-scale networks with demanding communication requirements. On a single FPGA, *Bluehive* can simulate 64,000 Izhikevich neurons in real-time. *NeuroFlow* (Cheung et al., 2016) is a platform that builds on top of Maxeler's^6^ Dataflow Engine (DFE) technology. A 6-FPGA system can simulate a network of 600,000 neurons. Real-time performance is achieved when simulating a network consisting of 400,000 neurons. The simulation of a plastic 1,000 neuron two-population Izhikevich model for 24 h biological time can be completed in 1,435 s, thus achieving a ~60-fold acceleration. The platform supports several neuron and synapse model types and a spike time dependent plasticity (STDP) rule. *NeuroFlow* also provides a PyNN interface (Davison et al., 2009)—a common Python interface for neural network simulators. In Wang et al. (2014) and Wang et al. (2018), an architecture is proposed that uses a procedural “on-the-fly” generation scheme for parameters and connections and is able to simulate 20 million to 2.6 billion leaky integrate and fire (IAF) neurons in real-time on a single Stratix V FPGA.

Such large scales come at a price and can only be achieved by accepting limitations regarding functionality, model complexity and simulation accuracy. These limitations may well represent acceptable trade-offs for the intended specific use cases, but can be severe with respect to the requirements of a platform for general neuroscience simulations. For example, in order to save hardware resources and reduce both computational costs and the amount of data to be processed, hardware implementations often use a large update interval of *h* = 1 ms to progress neuron model dynamics (e.g., Moore et al., 2012; Cheung et al., 2016; Wang et al., 2018). This is 10 times larger than the *de facto* standard used in digital simulations, and comes at the cost of numerical accuracy, especially for neuron models with stiff equations (Hansel et al., 1998; Morrison et al., 2007; Blundell et al., 2018b; Pauli et al., 2018). A further commonly-used trade-off with similar advantages and disadvantages is to represent neuron state variables in a low-precision fixed-point data format (e.g., Moore et al., 2012; Wang et al., 2018). It has been shown, for example, that the accuracy of the numerical integration of the Izhikevich neuron model dynamics is insufficient when a s16.15 representation, i.e., a 32-bit signed fixed-point data format is used (Gutzen et al., 2018; Trensch et al., 2018). Model complexity is reduced in the architecture proposed in Wang et al. (2014) and Wang et al. (2018) where individual synaptic connection delays are replaced by an axonal delay, thus avoiding the large memory structures and computational costs required to delay and accumulate incoming spike events.

These examples clearly demonstrate that it is challenging to reach design decisions that are simultaneously performant and flexible. The plethora of neuron and synapse models makes it difficult to come to design decisions that satisfy all requirements equally. There are also many questions relevant for the design which still lack an unambiguous answer and thus keep design decisions in a state of uncertainty. One example is the required numerical precision, which determines the specification of data types and the implementation of arithmetic operations—a design decision that effects implementation complexity, chip area and power efficiency. So far, only a few studies have examined the effects of numerical accuracy on simulation outcomes (e.g., Pfeil et al., 2012; Trensch et al., 2018; Dasbach et al., 2021).

Promising new design possibilities are also enabled by the integration on a single chip of FPGAs together with processor cores and other components to System-on-Chip (SoC) devices. This paves the way toward novel hybrid software-hardware approaches for application-specific implementations and new neuromorphic computing systems, such as the IBM Neural Computer INC-3000; a highly scalable parallel processing system. A single-cage system clusters 432 Xilinx Zynq SoC devices in a high bandwidth 3D mesh communication network (Narayanan et al., 2020). The system is highly flexible and applications can off-load algorithms and accelerate them using the programmable logic of the Zynq SoC devices. An example of such an application is the implementation of the cortical microcircuit model (Potjans and Diesmann, 2014) on the INC-3000 presented in Heittmann et al. (2022)—a reproduction of an equivalent NEST implementation and on the SpiNNaker neuromorphic system (cf. van Albada et al., 2018). The model consists of 0.8 · 10^5^ neurons and 0.3 · 10^9^ synaptic connections, was implemented in HLS, and utilizes 305 FPGAs. The simulation achieves an approx. four times speed-up compared with the biological time domain.

In this article, we introduce a novel SoC-based hybrid software and hardware mixed architecture approach for a neuromorphic compute node (henceforth *HNC node*) which is intended to work in a multi-node cluster configuration and capable of meeting the high demands for modeling and simulation in neuroscience. The development builds on the Xilinx Zynq-7000 SoC device architecture (Xilinx, 2021) and takes advantage of the tight coupling of a powerful FPGA device and a dual-core ARM Cortex-A9 processor core. The primary goal of the development is to provide a flexible platform for the accelerated simulation of neural network models which may consist of up to a few tens of thousands of neurons, a scale which covers the vast majority of current spiking neural network modeling studies. With the neuroscience requirement-driven design of the HNC node architecture, our development is to be seen as a complementary yet distinct approach to the neuromorphic developments aiming at brain-inspired and highly efficient novel computer architectures for solving real-world tasks.

We show that such a system can indeed be built, and that acceleration factors with respect to real-time in the order of 10–50 are realistically achievable for moderate workloads, with even higher factors possible for low workloads. We further demonstrate that the use of workload and performance models allow us to predict the performance characteristics of such a system under varying assumptions regarding workload and hardware design choices, some of which showing great potential as a substrate for neural simulations.

This article is organized as follows. Section 2 first gives an overview of the HNC node high-level architecture and the main design ideas. Section 3 presents the results of our performance measurements and an evaluation of the performance characteristics. A detailed presentation of the HNC node hardware and software architecture can be found in Section 4, with a focus on microarchitecture details critical to performance. In Section 5, we develop a workload and performance model to understand the performance characteristics of the HNC node and predict them for alternative assumptions in design space.

## 2. Overview of the Hybrid Neuromorphic Compute (HNC) Node

The HNC node architecture concept combines software-based and hardware-based implementations for the building blocks of a neural network simulation engine, and tightly couples both implementation types on a single chip; specifically, on a device of the Xilinx Zynq-7000 SoC family (Xilinx, 2021).

The underlying algorithms and the functional principle of the HNC node concept do not differ from those that are typically used in pure software implementations for time-discrete neural network simulations of point neuron models. It follows a hybrid strategy where neuron states are updated synchronously, time-driven, and at fixed intervals (e.g., Δ*t* = 0.1 ms) and synapses are updated asynchronously and event-driven, triggered when a synapse's presynaptic neuron emits a spike (Morrison et al., 2005).

While it is sufficient to implement performance non-critical tasks in software and let them be executed by general purpose processors, the performance-critical algorithms profit from mapping them to hardware. Non-critical tasks are, for example, the processes of node configuration, operation and simulation control, data type conversion, network instantiation, and user interaction. Critical to performance and simulation efficiency are the spike events processing and presynaptic data distribution, and the neuron and synapse model computations. The algorithms implemented in hardware bring the data and the operations performed on them close together and can thus alleviate problems which are inevitable with conventional systems, such as the von Neumann bottleneck.

**Figure 1:**
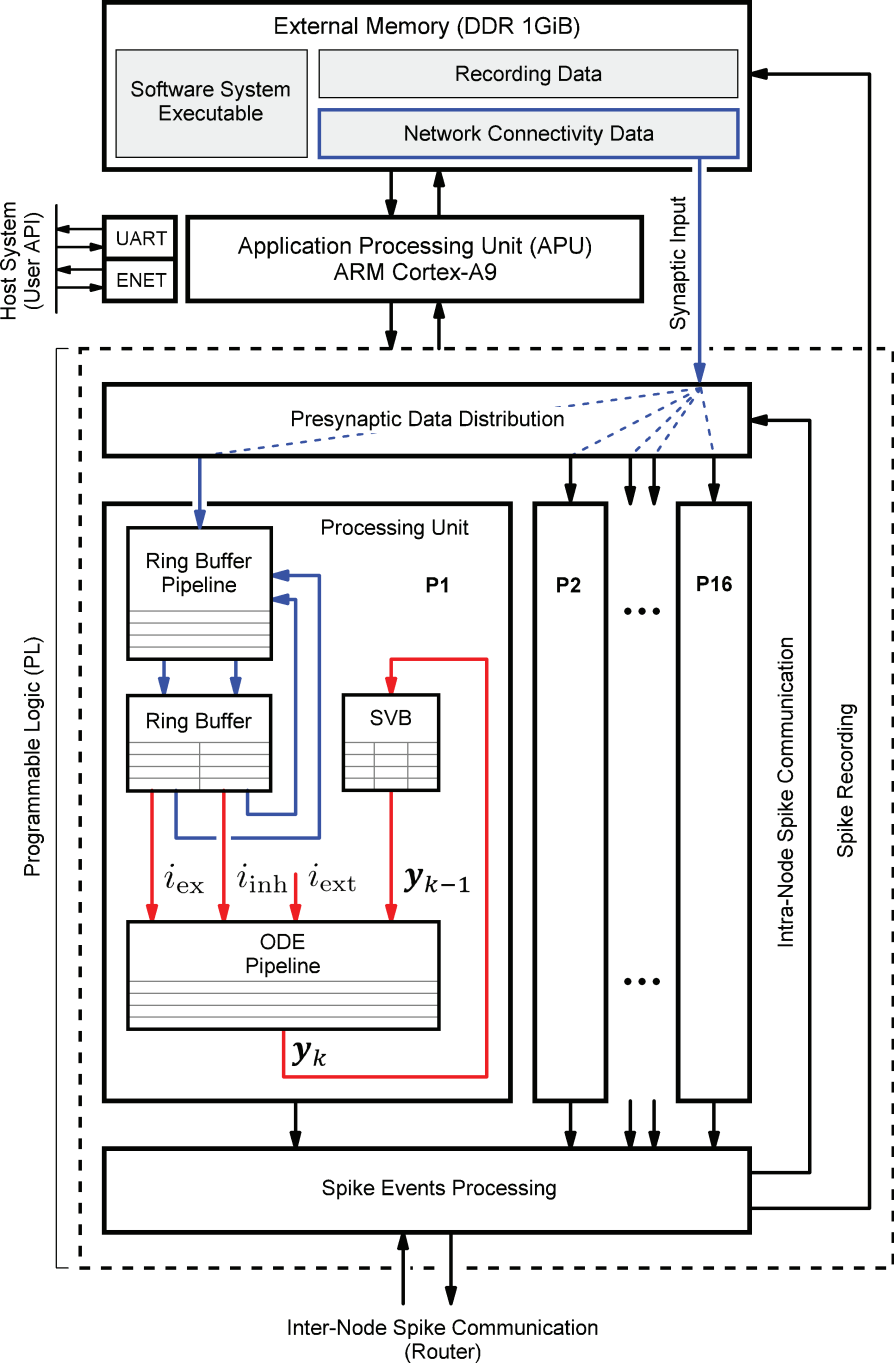
Hybrid neuromorphic compute (HNC) node high-level architecture. The highest architectural level of the HNC node comprises three main components: an off-chip external memory (top), an application processing unit (APU; middle), and a programmable logic part (PL; lower dashed box). In order to distribute the workload and parallelize operations, the PL implements 16 identical processing units (P1, P2,.., P16). The red and blue arrows indicate two distinct processes that are critical to performance and primarily determine the performance characteristics and achievable acceleration factors. Red arrows: the process of the neuron and synapse model state update performed by the ordinary differential equations solver pipelines (ODE pipelines) which operate on fast on-chip block RAM memories that constitute the state variables buffer (SVBs). Blue arrows: the process of the presynaptic data distribution and processing which hold the data it operates on in the slow external off-chip memory.

Figure 1 shows the HNC node high-level architecture concept, which consists of three main components: (i) an off-chip external memory (top); (ii) an application processing unit (APU; middle); and (iii) a programmable logic part (PL; dashed box). A more detailed description of the high-level system architecture and the microarchitecture is given in Section 4.1.

Both the APU and the PL are connected to the off-chip external memory. It contains the node control software (Section 4.2) which is executed by the APU orchestrating the overall node operation, and also holds the node-local connectivity data of the neural network being simulated and buffers the recorded spike data. Storing the connectivity data in a slow, external memory is one of the decisive performance limiting factors of the system. This aspect is discussed in detail Section 4.3.1. However, there are two important factors leading to this design decision. The first is a functional requirement: even though the current development does not yet include plasticity, in order to be able to cope with synaptic and structural plasticity algorithms in future, the synaptic connections must be stored, accessible, and changeable. In contrast, for static networks, performance-efficient solutions have been developed which makes use of a procedural connectivity generation approach (Knight and Nowotny, 2021; Heittmann et al., 2022) where the synaptic connections are determined algorithmically during the simulation, thus avoiding having to retrieve them from memory. The second is a resource constraint due to technical limitations of the technology: fast, low-latency, on-chip block RAM (BRAM) would be ideal to hold this data, but BRAM is a limited FPGA resource and the memory requirement for storing a network's connectivity data is demanding. For example, given a 64-bit data item to represent a single connection, a natural dense network, such as the cortical microcircuit model (Potjans and Diesmann, 2014) comprising 0.8 · 10^5^ neurons and 0.3 · 10^9^ connections requires 2.4 Gbyte of memory in total. That is 24 Mbyte per compute node if a single node processes 10^3^ neurons. The Xilinx Zynq-7000 SoC device used in this work provides only 19.2 Mbit of BRAM, i.e., 10 times less than required.

The PL, i.e., the FPGA part of the SoC, implements 16 identical hardware processing units (P1, P2,.., P16). Each is capable of carrying out the computations for *N*^*P*^ = 64 neurons. This allows a total of *N* = 1,024 neurons to be processed on a single chip or HNC node, respectively. The PL and APU are closely coupled through high performance streaming and memory mapped interfaces which allow an efficient data exchange between the two parts. The PL is also directly connected to the off-chip external memory, thus enabling APU-independent memory read and write operations.

Each processing unit processes its 64 neurons in a pipeline fashion, updating the neuron states at fixed intervals of Δ*t* = 0.1 ms. The neuron states ***y***_*k*_ are thereby held in state vector buffers (SVB) which are implemented as fast block RAM (BRAM) memories on the PL. The associated data paths of this time-driven process are indicated in Figure 1 by the red arrows.

The blue arrows in Figure 1 mark the data paths involved in the event-driven presynaptic data processing. The post-synaptic spike events (up to 16 spike events can occur in parallel at a time; one per processing unit) are serialized and packed for communication and recording. This is handled by the spike events processing module. If a spike event occurs, it initiates read operations from external memory to obtain the network's connectivity data, i.e., the node-local synaptic connections of the firing neuron, from which the synaptic inputs are derived. The presynaptic data distribution module parallelizes this data and delivers the synaptic inputs to the processing units (P1, P2,.., P16); this is indicated by the dashed blue lines in Figure 1, thereby distributing the workload generated by the incoming presynaptic spike events. The ring buffers (RB) implement the synaptic transmission delays and store the accumulated synaptic inputs, i.e., the lumped excitatory *i*_ex_ and inhibitory *i*_inh_ values. Since the number of synapses by far predominates, the whole process of presynaptic data distribution and processing is critical to performance.

## 3. Results

### 3.1. Single Node Performance

In the following, we consider an isolated HNC node that is not embedded in a multi-node system for which otherwise inter-node communication and synchronization latencies cannot be ignored. For an isolated node, the previously explained two distinct processes will exclusively determine performance where the neuron state update process (red arrows in Figure 1), and the process of presynaptic data distribution and processing (blue arrows in Figure 1), contribute to different performance relevant aspects. In Section 5.2, a performance model is presented that is based on the HNC node microarchitecture implementation details explained in Section 4.3. By additionally taking communication latencies, inevitably occurring in a multi-node system, into account, the model will also allow conclusions to be drawn about the acceleration factors achievable for larger network sizes and workloads.

The current HNC node design implements *N*^M^ = 1024 neurons and allows *C*^M^ = 128 target connections per source neuron and node. This is in agreement with a connection probability value of approx. ϵ = 0.1 observed in Braitenberg and Schüz (1998). Note that the possible number of a source neuron's target connections is not restricted to the value of *C*^M^. It scales linearly with the number of HNC nodes *M* in a cluster, i.e., it yields *MC*^M^. A typical cortical neuron connects to between 1,000 and 10,000 other neurons. Consequently, a network of *N* = 10^5^ where each neuron has 10^4^ connections represents an upper limit with regard to memory requirements and workload; beyond this, the total number of synapses in a network scales linearly rather than quadratically.

In order to evaluate the HNC node's capability to perform in different workload situations, we investigate a two-population network model consisting of 1,000 neurons (see Section 5.3). We measure the time to simulate the network and calculate the acceleration factor as the quotient of the measured simulation duration in wall clock time and the simulated biological time of 300 s. We systematically vary the external input current from *i*_ext_ = −3.0 pA to 100.0 pA. The increasing external offset current causes the network to run through a wide range of activity, from quiescence up to an average firing rate of $\bar{\nu }=300\text{ spks}/\text{s}$ and thus an increase in the workload. According to the workload model described in Sec. 5.1, this results in an average number of spike events per simulation time step (*h* = 0.1 ms) ranging from ${\bar{\nu }}_{\text{k}}=0$ to ${\bar{\nu }}_{\text{k}}=30$.

**Figure 2:**
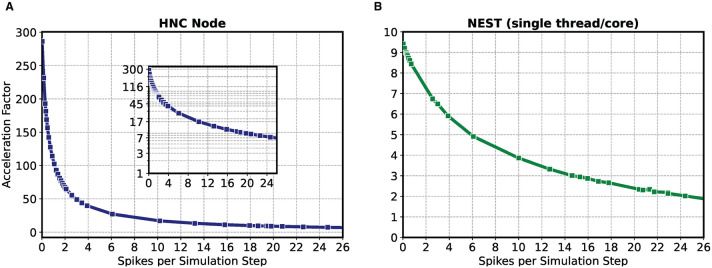
Performance as a function of workload for the HNC node and NEST. The acceleration factor (wall clock time divided by the biological time) as a function of the average number of spike events per simulation time step ${\bar{\nu }}_{\text{k}}$ of the HNC node using a PL clock frequency of *f*_clk_ = 200 MHz **(A)** and the neural simulation tool NEST on an Intel(R) Core(TM) i7-7700K CPU 4.20 GHz (Kaby Lake architecture) **(B)**. The measurements were carried out with *h* = 0.1 ms simulation resolution. In consecutive simulation runs of 5 min simulated biological time, the 1,000 neuron two-population Izhikevich neural network model described in Section 5.3 was stimulated with an increasing external offset current *i*_ext_ = {−3.0 pA, .., +100 pA}. Inset in **(A)** gives a log-lin representation.

The result of the HNC node performance measurement is shown in Figure 2A. For comparison, Figure 2B shows the results for the same model implemented in NEST 2.20.1 (Fardet et al., 2020) and executed on an Intel(R) Core(TM) i7-7700K CPU 4.20 GHz (Kaby Lake architecture). If the workload is in the range of a few spike events per simulation time step, the HNC node outperforms the NEST simulation on the Intel Kaby Lake CPU, and this even at ~4.5 W power consumption (see the power report given in the Supplementary Material)—with the Intel Kaby Lake CPU, a power consumption of several tens of Watts is to be expected. If the external current is set to zero, the network fires with an average rate of $\bar{\nu }=7.0\text{ spks}/\text{s}$, which corresponds to a number of spikes per time step of ${\bar{\nu }}_{\text{k}}=0.7$. For this workload, the acceleration factor achieved for the NEST simulation is 8.4 compared to a factor of 127.0 measured for the HNC node. The NEST simulator used for the comparison is a runtime-optimized and flexible tool for a wide range of neural network simulations and as such, is a good reference in this regard. Clearly, a CPU-optimized implementation of the specific network model can achieve even better results^7^. However, the difference in performance and efficiency is such that the HNC node performance is beyond the reach of any CPU implementation. At low workloads, the hardware implementation can fully utilize its capabilities. Pipelining and the parallelization of operations increases throughput and reduce latencies. This is mainly to be ascribed to the process of the neuron state update, indicated by the red arrows in Figure 1. Its implementation benefits from data-locality that is achieved by storing variables in fast, low-latency on-chip BRAM memories.

As the workload increases, the NEST implementation undergoes a moderate degradation in performance. In contrast, the performance deteriorates rapidly on the HNC node. This is a trivial consequence of the data access latency and limited bandwidth of the external memory decelerating the process of the pre-synaptic data distribution and processing (marked by the blue arrows in Figure 1), which now dominates operation. This is examined in greater detail below for different hardware design choices. Moreover, the measurements of the single HNC node and CPU core performances only give an upper baseline. For the simulation of larger networks on multi-node or many-core systems in the following we examine the effect on performance of the additional latencies arising from synchronization and communication.

### 3.2. Performance Characteristics

**Figure 3:**
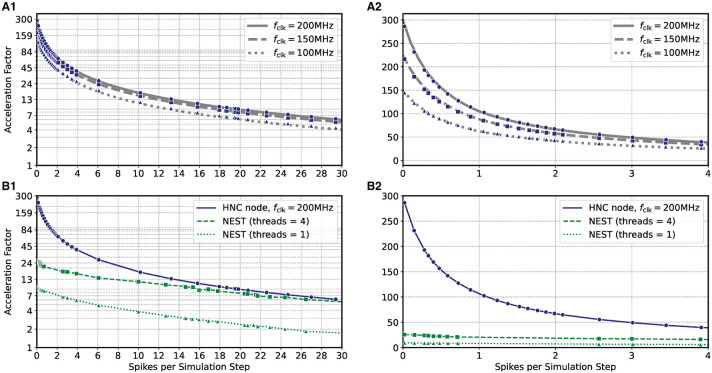
Performance as a function of PL clock frequency and workload for the HNC node and NEST. **(A)** Measured acceleration factors of the HNC node (blue markers) as a function of workload for three different clock frequencies in log-lin **(A1)** and linear **(A2)** representation. Gray curves show the predictions of the performance model (Section 5.2). **(B)** As in **(A)**, but comparing the performance of the HNC node running at a PL clock frequency of *f*_clk_ = 200 MHz to that of a NEST implementation using one or four threads on an Intel(R) Core(TM) i7-7700K CPU 4.20 GHz.

Based on the HNC node microarchitecture (Section 4.3) and their operating latencies (Section 4.3.3) a performance model is developed in Section 5.2. This model is used in the following to evaluate the performance characteristics of the HNC node as a stand-alone compute node and when operating in a cluster configuration. In order to verify the correctness of the performance model, we repeated the measurements carried out in the previous section using three different PL clock frequencies *f*_clk_ = 100/150/200 MHz. The results are shown in Figures 3A1,A2 where the blue markers indicate the measured acceleration factors and the gray curves are calculated from Equation (7) of the performance model. The predicted acceleration factors are in almost perfect agreement with the measured values.

The results also reveal that as the workload increases, the achievable acceleration factor is increasingly determined—and thus limited—by external memory access times (i.e., by the term ${\bar{\nu }}_{k}L_{\text{DS}}$ in Equation 7). This can not be compensated by a higher PL clock frequency. However, an acceleration factor of ~100 is achieved for moderate workloads, i.e, ${\bar{\nu }}_{\text{k}}\approx 1$, *h* = 0.1 ms. Such a workload is created, for example, by a network consisting of *N* = 5,000 neurons with an average firing rate of $\bar{\nu }\approx 2\text{ spks}/\text{s}$.

Figures 3B1,B2 compares the HNC node measurements at a PL clock frequency of *f*_clk_ = 200 MHz to the equivalent simulation in NEST on a four-core Intel CPU. At low workloads, the HNC node is an order of magnitude faster than the NEST/CPU implementation. Even at high workloads, the HNC node still simulates substantially faster than a single state-of-the-art processor core. Such high workloads are not only of theoretical interest in benchmarking tasks. As ${\bar{\nu }}_{\text{k}}$ increases linearly with the network size *N* (Section 5.1), from a single-node workload perspective and assuming a fixed number of neurons per node, a small network at high average firing rates is equivalent to a large network utilizing multiple nodes and exhibiting a low average firing rate.

For example, for the cortical microcircuit model (Potjans and Diesmann, 2014) which consists of *N* ≈ 0.8 · 10^5^ neurons, a value of ${\bar{\nu }}_{\text{k}}\approx 23$ can be expected^8^. At this workload the HNC node achieves an acceleration factor of ~7 while for a single-threaded NEST simulation a factor of ~2 was measured. If the NEST workload is distributed, in the sense of strong-scaling utilizing all four cores of the Intel CPU, the NEST simulation is nearly as fast as the HNC node. Note that ${\bar{\nu }}_{\text{k}}\approx 23$ is a theoretical value in this case, as the current single node implementation cannot accommodate a network as large as the cortical microcircuit model.

Even though power efficiency was not considered in this work, it is worth mentioning that the SoC device's power consumption is in the order of just a few Watts, and thus achieves a much higher simulation efficiency than the Intel core.

If the HNC node is to be operated in a cluster, the adverse effect that additional inter-node communication has on performance could influence design decisions such as the number of neurons per node and processing unit. For illustration, we consider four sets of design parameters. These are as follows: **prototype**—the parameter set corresponding to the prototypical implementation generating the measurements presented above, implementing *P* = 16 processing units, *DS* = 2 data streams (marked S1 and S2 in **Figures 8**, **9**), and *N*^P^ = 64 neurons per ODE pipeline; **high data stream parallelism**—as for *prototype* but assuming that each processing unit connects to its own data stream (*P* = 16, *DS* = 16, *N*^P^ = 64) introducing a factor eight times reduction of external memory access latency; **high processing units parallelism**—as for *high data stream parallelism* but implementing twice the number of processing units in order to halve the ODE pipeline iteration latency and increase the maximum achievable single-node acceleration factor (*P* = 32, *DS* = 16, *N*^P^ = 32); and **low processing units parallelism**—the opposite of *high processing units parallelism*, reducing the number of processing units (*P* = 8, *DS* = 16, *N*^P^ = 128). The parameter sets are shown in Table 1. Note that the parameter sets, with the exception of the *prototype* configuration, have not been applied to the HNC node. The SoC device selected for this study is limited to the *prototype* configuration in terms of number of data streams.

**Table 1:** Parameter sets.

**Parameters**	**Prototype**	**High** **data stream** **parallelism**	**High** **proc. units** **parallelism**	**Low** **proc. units** **parallelism**
Number of data streams, *DS*	2	16	16	16
Data stream latency, *L*_DS_	110	14	14	14
Number processing units, *P*	16	16	32	8
Number of neurons per processing unit, *N*^P^	64	64	32	128
ODE pipeline iteration latency, *IL*_N_	64	64	32	128
**Acceleration factors w/o communication**				
Maximum, $F_{\text{S}}^{\text{MAX}}=F_{\text{S}}\left({\bar{\nu }}_{\text{k}}=0\right)$	298.5	298.5	571.4	152.7
Low workload, *F*_S_(1.0)	104.7	177.0	246.9	113.0
Medium workload, *F*_S_(10.0)	16.9	84.5	97.7	66.5
High workload, *F*_S_(20.0)	8.8	52.4	58.4	45.6
**Acceleration factors with communication**				
Maximum, $F_{\text{C}}^{\text{MAX}}=F_{\text{C}}\left(0\right)$	119.8	119.8	148.1	86.6
Low workload, *F*_C_(1.0)	67.6	91.7	107.5	70.9
Medium workload, *F*_C_(10.0)	15.0	51.7	56.4	44.4
High workload, *F*_C_(20.0)	8.1	34.8	36.9	31.3

*The acceleration factors are calculated using the performance model (Section 5.2) for four different parameter sets *prototype*; *high data stream parallelism*; *high processing units parallelism*; *low processing units parallelism*, and for three different workload situations, *low, medium*, and *high* as well as with and without inter-node communication. The number of neurons per node *N*^M^ = *PN*^P^ = 1024, the PL clock frequency *f*_clk_ = 200 MHz, the transmission latency time *T*_COM_ = 500 ns, and the per spike event transmission latency factor α = 0.05 (see main text and Section 5.2 for description) are the same for all parameter sets*.

The number of neurons per node (*N*^M^ = 1024) and the PL clock frequency (*f*_clk_ = 200 MHz) are kept constant across the parameter sets. To describe the effect of inter-node communication on performance, the performance model developed in Section 5.2 introduces two parameters: the transmission latency time *T*_COM_, and the per spike event transmission latency factor α (for a description of the parameters see Section 5.2). Their values were set to *T*_COM_ = 500 ns and α = 0.05. They are the same for all parameter sets. The choice for the transmission latency time is motivated by the temporal resolution of *h* = 0.1 ms and an envisioned acceleration factor of 100, which would be a major breakthrough for reproducible large-scale neuroscience simulations. This assigns *T*_COM_ half of the wall clock time that would be available to complete a single simulation step. The value of the per spike event transmission latency factor was arbitrarily chosen and corresponds to 5 additional clock cycles per spike event at a given PL clock frequency of *f*_clk_ = 200 MHz.

**Figure 4:**
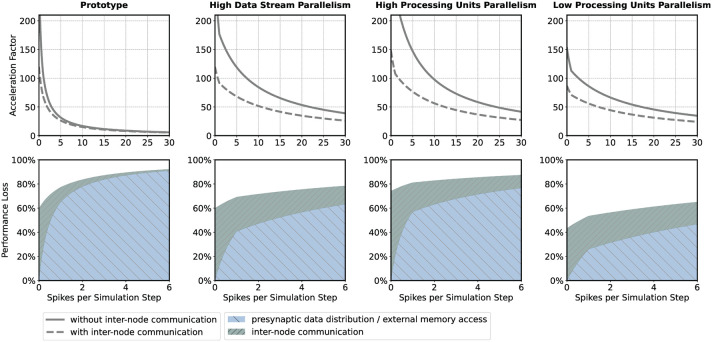
Performance characteristics estimation. Performance characteristics of the HNC node are calculated using the performance model (Section 5.2) for the parameter sets *prototype*; *high data stream parallelism*; *high processing units parallelism*; *low processing units parallelism*. See main text and Table 1 for details. The upper panels show the achievable acceleration factors as a function of workload with inter-node communication $F_{\text{C}}\left({\bar{\nu }}_{\text{k}}\right)$ (dashed curves) and without inter-node communication $F_{\text{S}}\left({\bar{\nu }}_{\text{k}}\right)$ (solid curves); the lower panels show the stacked plots of the respective contributions to the loss of performance with respect to the maximum achievable single-node acceleration factor $F_{\text{S}}^{MAX}$ of the inter-node communication $P_{\text{C}}\left({\bar{\nu }}_{\text{k}}\right)$ (green) and presynaptic data distribution $P_{\text{S}}\left({\bar{\nu }}_{\text{k}}\right)$ (blue) (see Section 5.2).

The upper panels in Figure 4 show the acceleration factors as a function of the workload calculated according to the performance model (Section 5.2, Equations 7, 8) both with and without inter-node communication. In addition, the lower panels in Figure 4 provide an alternative representation of the curves, namely as the respective proportion of performance loss (with respect to the maximum achievable single-node acceleration factor for the corresponding parameter configuration) caused by inter-node communication and by the process of the presynaptic data distribution—mainly the effect of external memory access latency (Section 5.2, Equations 11, 12). Table 1 shows the calculated acceleration factors for low, medium, and high workload.

As one would expect, the additional communication latency reduces the maximum achievable acceleration factors. For the *prototype* configuration (Figure 4, prototype, upper panel), for example, the factor decreases from 298.5 to 119.8 (Table 1). As the workload increases, the effect becomes progressively smaller. For the *prototype* configuration, for low workload, the factor decreases by 35.5%, for medium workload by 11.2%, and for high workload by 7.9%. For low workload, the achievable acceleration is now determined by inter-node communication latency, but toward higher workload external memory access time is still the main contributor to performance degradation (Figure 4, prototype, lower panel).

In the *high data streaming parallelism* configuration, we therefore assign each processing unit its own data stream, and by this means, introduce eight times higher parallelism in the presynaptic data distribution—the two data streams S1 and S2 (**Figure 8**) are each split into eight streams, thus reducing external memory access times by a factor of eight. Figure 4 (high data stream parallelism, upper and lower panel) illustrate the effect. For medium workload and with inter-node communication, the acceleration factor increases from 15.0 (for the prototype configuration) to 51.7, i.e., by a factor of 3.4.

One may try to further improve performance by an increase in the parallelism of the neuron and synapse model processing, i.e., by introducing a higher number of processing units. The *high processing units parallelism* configuration doubles the number of processing units. This configuration achieves a very high maximum acceleration factor of 571.4 for the single node without inter-node communication. In a cluster such high acceleration cannot be realized, even for low workload. Bound by inter-node communication latency, the performance loss in relation to the maximum acceleration is 74%, and for low workload 81.2%. However, for high workload, external memory access time is still the main limiting factor (Figure 4, high processing units parallelism, upper and lower panel).

With regard to the hardware footprint and the required FPGA resources—which is an important aspect of hardware designs—the effect of a reduction of the number of processing units is also of interest. The *low processing units parallelism* configuration, therefore, implements half of the processing units of the *prototype* configuration (Figure 4, low processing units parallelism, upper and lower panel). For low workload and in comparison to the *high processing units parallelism* configuration, the acceleration factor decreases from 107.5 to 70.9, i.e., by 34%. For high workload, the acceleration factor decreases from 36.9 to 31.3. This is a loss of only 15.2% and might be an acceptable degradation when making design decisions oriented toward a high workload scenario, given that thereby 75% of ODE pipeline hardware resource, namely digital signal processing (DSP) units, can be saved with this configuration. Saving hardware resources reduces power consumption and thus increases simulation efficiency. Considering the above, for medium workload the *high data stream parallelism* configuration can be a compromise with regard to the achievable acceleration factors for different workload situations and the required chip resources. For the HNC node prototype implementation the utilization of the SoC chip resource are given in the Supplementary Material.

The current implementation of the HNC node configured with the *prototype* parameter set and operated in a cluster would achieve an acceleration factor in the order of 10–50 for medium and small workloads. Such a workload is created, for example, by a network consisting of *N* = 10,000 neurons with an average firing rate of $\bar{\nu }\approx 2..10\text{ spks}/\text{s}$. To simulate such a network, 10 HNC nodes would need to be clustered.

### 3.3. Correctness

In order to meet the requirement of an accurate and reproducible simulation we evaluated the equivalence of the simulation results produced by the HNC node and a ground truth. In this validation process we aimed for the reproduction of the dynamics of a selected network state obtained from a reference implementation of the two-population Izhikevich network described in Section 5.3. This reference implementation was written in the C language and developed as part of an earlier study (Trensch et al., 2018). The source code is available online^9^. To create the network state, the ground truth, the network was trained for 1 h biological time using a spike time dependent plasticity (STDP) rule (see the description of the network given in the Supplementary Material). After 1 h of simulated network time, the current state of the network was captured by exporting the network's connectivity data. The connectivity data was then imported back into the C simulation, and with the STDP rule turned off, from 30 min simulated time the spikes were recorded while the network was stimulated with a random input. This recorded network activity data defined the ground truth, that is, the captured network state that defines a reliable reference. For reproduction, we loaded the connectivity data into the HNC node and repeated the simulation. To provide further evidence and to substantiate the correctness of the simulation result generated by the HNC node, the connectivity data was also imported into the NEST simulator and we repeated the simulation again. When simulating a network, it is sufficient to communicate spike events at intervals less or equal to the minimum synaptic delay in the network. The NEST implementation makes use of this and propagates spike events on a 1 ms grid - the minimal synaptic delay in the two-population Izhikevich network. In contrast, the HNC node communicates spike events at 0.1 ms intervals. For progressing neuron model dynamics, an integration step size of *h* = 1 ms would not be sufficient to achieve the necessary numerical accuracy (Pauli et al., 2018). Therefore, both NEST and the HNC node use an integration step size of *h* = 0.1 ms. The NEST Izhikevich neuron model implementation was adapted accordingly. The simulation script and the source code is available online^9^.

**Figure 5:**
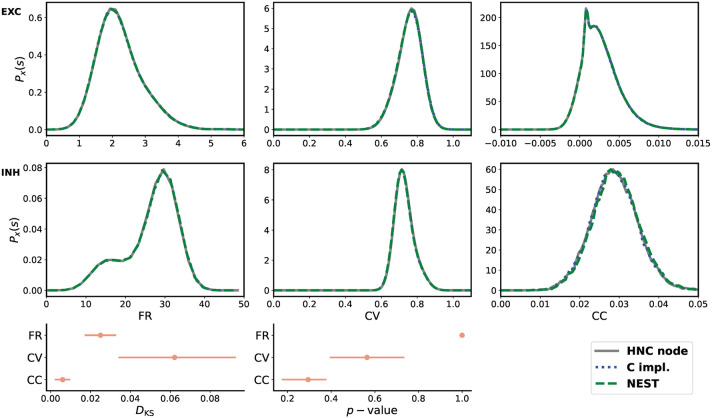
Quantitative comparison of statistical measures. Upper two rows from left to right: probability distribution of average firing rate (FR), coefficient of variation (CV), and Pearson's correlation coefficient (CC) for the excitatory (EXC) and the inhibitory (INH) population. The measures were calculated from 30 min simulated time. For the calculation of CC, spike trains were binned at 2 ms. In order to derive the probability distributions from the calculated measures, the Freedman-Diaconis rule was applied to select the width of the bins of the distribution histograms, and a Gaussian kernel was used for density smoothing. The bottom row shows the Kolmogorov-Smirnov statistics calculated from the raw samples of the calculated statistical measures. During the simulations performed on the HNC node, using the NEST simulator, and carried out using the reference C implementation, the network was stimulated with a different random input—a limitation of the HNC node prototype and hardware implementation of the PRNG. All three simulations used the same explicit Forward Euler integration method with an integration step size of *h* = 0.1 ms. All measures are in close agreement and show statistical equivalence.

From the three obtained data sets of network activity, the probability distribution of the firing rates (FR), the coefficient of variation (CV), and the Pearson's correlation coefficient (CC) were calculated and compared. The statistical measures are described in the Supplementary Material. The result of the comparison is shown in Figure 5. All measures are in close agreement and show statistical equivalence.

Simulation results must not only be reproducible and in agreement with a reliable reference, but also replicable, i.e., spike-identical in repeated simulations. Replicability was tested by repeatedly simulating the two-population Izhikevich network for 20 minutes simulated time. Due to limited numerical precision and rounding errors, operations are not commutative. Therefore, and to strengthen the tests, the network was also logically shifted across processing units in order to assign a logical neuron-id to different hardware resources, and thus force a different spike ordering and scheduling of operations. The simulation results were successfully validated for spike-identicality (data not shown).

## 4. Architecture

### 4.1. System-Level Architecture

We chose the XCZ7045 SoC from the Xilinx Zynq-7000 SoC device family (Xilinx, 2021) for the technical implementation, and all work presented in this article was carried out on a Xilinx Zynq-7000 SoC ZC706 development board (Xilinx, 2019e). The XCZ7045 integrates a dual-core ARM Cortex-A9 processor (up to 1 GHz) and a freely programmable and re-configurable logic device, i.e., an FPGA with the size of 350,000 configurable logic blocks (CLBs). It provides ~218,000 look-up tables (LUTs), ~437,000 flip-flops (FFs), 19.2 Mbit of fast static block RAM (BRAM) that can be customized for different configurations, and 900 digital signal processing (DSP) blocks for the implementation of arithmetic operations.

**Figure 6:**
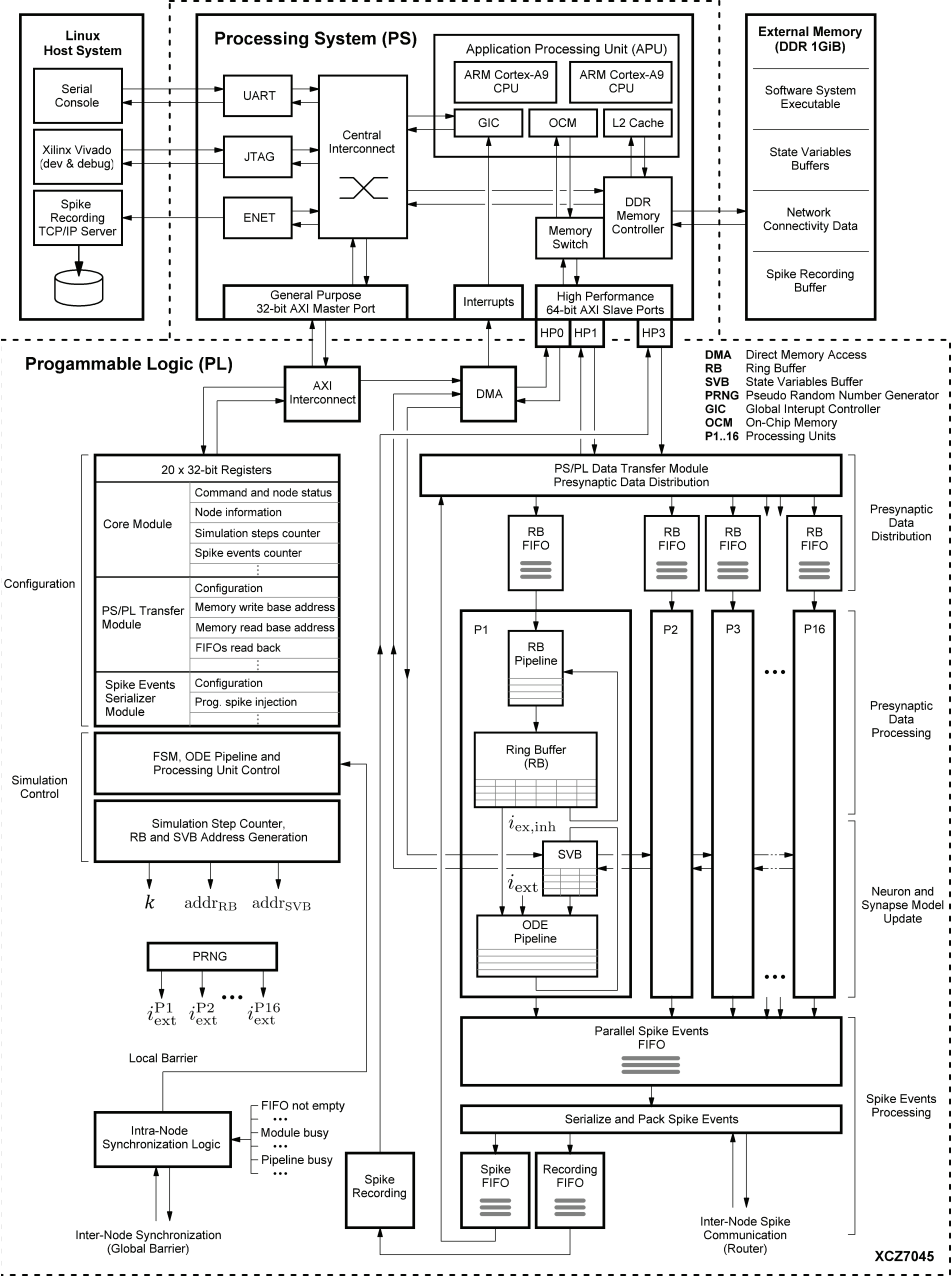
System-level view of the HNC node hardware architecture. The on-chip components are framed by the dashed lines. The lower frame encloses all modules that have been implemented in programmable logic (PL), while in the upper frame the components of the processing system (PS) are shown. Attached to it is an external 1 GiB DDR RAM module (upper right). It stores the node software system executable and the data structures required for operation, for example, the state variables and connectivity information. The external memory also functions as buffer for the recorded spike data. The PS is further connected to a Linux host system (upper left) which provides a serial console to operate the HNC node, the Xilinx Vivado environment for development, and a TCP/IP server to collect the recorded binary spike data.

Figure 6 shows the system-level view of the implemented HNC node architecture. It details the major components and modules, their interaction and functional assignments. The operation of the HNC node is software-controlled. The program executable is located in the external memory (top right) and executed by the processing system's (PS) application processing unit (APU) (upper dashed box). For user interaction, debugging and data exchange, the HNC node is connected to a Linux host system (upper left) *via* an Ethernet (ENET) connection for the read-out of recorded spike events, a JTAG^10^ connection for programming and debugging, and a serial UART^11^ user console interface.

The simulation engine's core components are realized in programmable logic (PL). They are shown in the lower dashed box in Figure 6. Function-wise, the hardware components can be assigned to four distinct steps in the process of carrying out a simulation cycle: (i) presynaptic data distribution; (ii) presynaptic data processing; (iii) neuron and synapse model update; and (iv) spike events processing.

*Presynaptic data distribution*: triggered by postsynaptic spike events, the *PS/PL Data Transfer Module* initiates read operations from the external memory to obtain the node-local connectivity information (see Section 4.3.1) of the firing neurons. In order to do so and make optimal use of the read bandwidth of the external memory, the *PS/PL Data Transfer Module* is connected to the PS *via* a pair of high performance ports (HP1, HP3) capable of working independently of one another. At its outputs, the module connects to a series of first-in-first-out (FIFO) buffers (in Figure 6 referred to as RB FIFOs) which compensate for latencies and to which the presynaptic date is distributed. The RB FIFOs connect the *PS/PL Data Transfer Module* to 16 identical processing units (P1, P2,.., P16). The processing units parallelize and pipeline the computations for the presynaptic data processing and the neuron and synapse model dynamics.

*Presynaptic data processing*: In order to derive the synaptic inputs *i*_ex_ and *i*_inh_ from the presynaptic data, the presynaptic data is fetched from the RB FIFOs and passed through the RB pipelines. The RB pipelines operate on the ring buffers (RBs) and accumulate the synaptic inputs, the values of which are stored and delayed for further processing by placing them into the RBs.

*Neuron and synapse model update*: The ordinary differential equation solver pipelines (ODE pipelines) retrieve the accumulated synaptic input values from the RBs and progresses the neuron and synapse model dynamics; updating the models' state vectors in the state variables buffers (SVBs). In addition, an XNOR-shift PRNG can provide a random external network stimulus $\left\{i_{\text{ext}}^{\text{P}1},..,i_{\text{ext}}^{\text{P}16}\right\}$ which is directly applied to the neurons in the ODE pipelines.

*Spike events processing*: In principle, there can be as many spike events occurring in each unit, and in a single simulation time step *k*, as the number of neurons processed in a pipeline. In other words, in extreme, 16·*N*^P^ = *N*^M^ = 1,024 spike events need be buffered, serialized and packed for local (intra-node) and external (inter-node) spike communication, as well as for recording. The associated components that are related to this process are shown at the lower right in Figure 6.

In order to enable the APU to perform software-controlled read and write operations on the SVBs to access the state variables, all processing units are chained to one another and connected to a direct memory access (DMA) controller.

The aforementioned modules mainly represent the data paths or operate on them. To orchestrate the control flow, additional components are required for configuration, simulation control, and synchronization. For configuration and simulation control, a bank of 32-bit registers store node control and status information (shown at the mid left in Figure 6). All registers are mapped into the APU's address space and thus accessible by the node software. Their settings steer the operation of a finite state machine (FSM) responsible for generating all control signal sequences for the different operating modes (e.g., load state variables, progress simulation by *k* steps, unload state variables). To preserve the temporal causality and ensure the correct sequence of operations, all spike events of a simulation step *k* must have been delivered and the RB buffer updates must have been completed before the next simulation step *k* + 1 can be initiated. This is ensured by an intra-node synchronization logic which monitors the operating status of all modules. The module is shown at the lower left in Figure 6. Technically, it implements a barrier mechanism that synchronizes the overall processing at the end of every simulation step. In a multi-node configuration this extends to an inter-node barrier message—software simulators, such as NEST (Gewaltig and Diesmann, 2007) use MPI^12^ barrier calls for this purpose.

The entire hardware design—with the exception of the DMA controller and the FIFO blocks for which Xilinx soft IP cores were used—was implemented on the register transfer level (RTL) in VHDL. The decision to take this more arduous and time consuming approach—rather than a high level synthesis (HLS) implementation (Xilinx, 2019c)—is motivated by the endeavor to maximize control over the microarchitecture details in order to optimize the timing behavior. The current HNC node implementation works stable up to a PL clock frequency of *f*_clk_ = 200 MHz. The software implementation was carried out in the C language. For the development process the Xilinx Vivado Design Suite (Xilinx, 2019d) and the Xilinx Vivado SDK and embedded system tools (Xilinx, 2019b) were used which provide the development tools for hardware-software co-design, synthesis and analysis.

### 4.2. Software System Architecture

**Figure 7:**
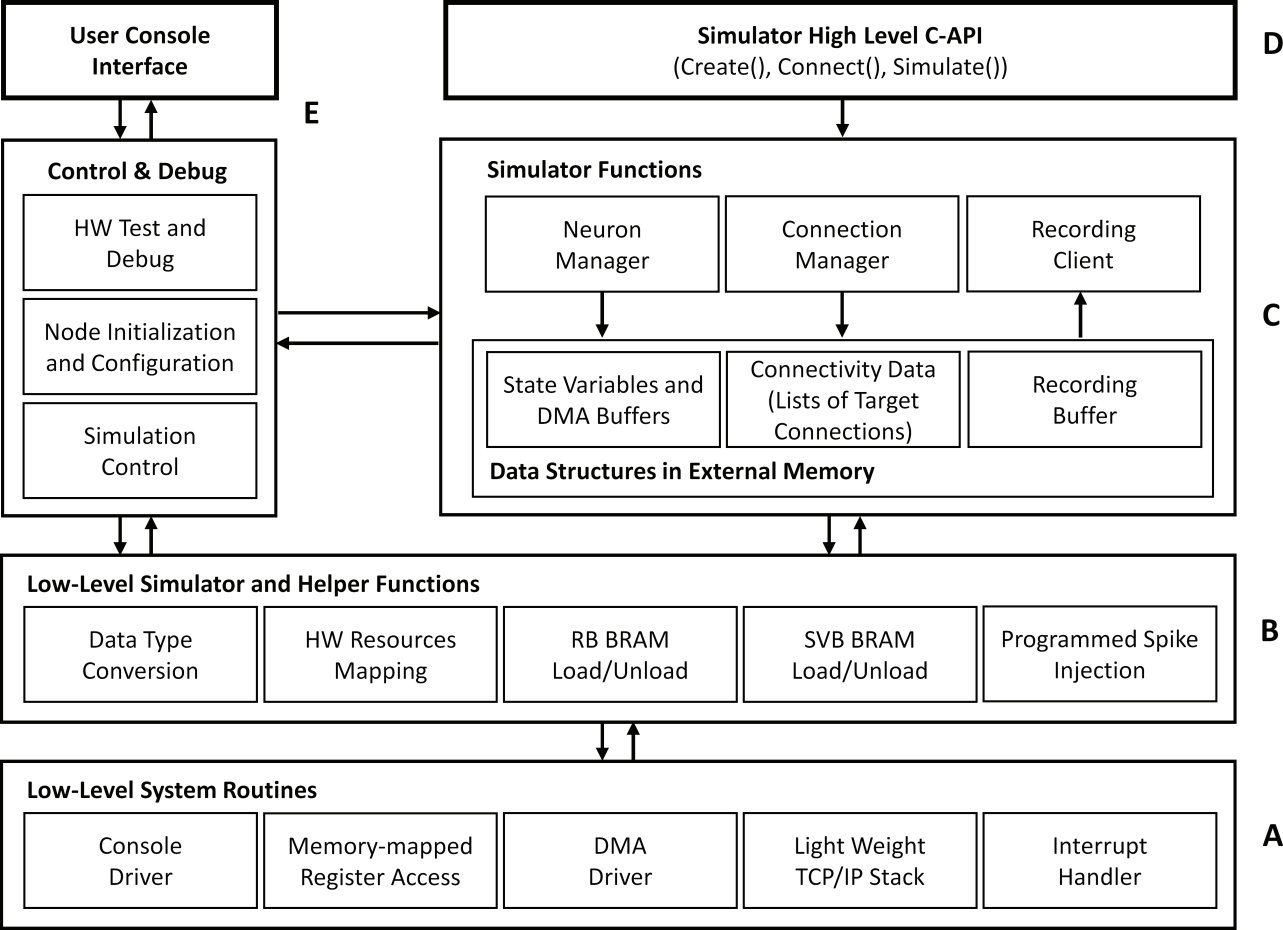
HNC node software system architecture. The tiered architecture provides abstraction at different functional levels. **(A)** The low-level system routines hide technical details about the operation of the implemented hardware components. Based on this, low-level simulator and helper functions **(B)** form the foundation for the core component of the HNC node software **(C)**, that is, the simulator functions. **(D)** At the highest level, a minimal set of functions is provided to instantiate and simulate a network. **(E)** In addition, the software system implements components that are responsible for control, testing, and debugging and also enable user interaction.

Figure 7 outlines the basic architecture of the HNC node software system, which is executed by the SoC's integrated APU. At its lowest level, an abstraction layer provides fundamental routines to drive the hardware functions, for example, to reset and initialize components, to handle interrupts, to establish a basic serial console and TCP/IP communication, and to initiate direct memory access (DMA) transfer operations. Helper- and low-level simulator functions, such as routines to load and unload the state variable buffers, build on top of this layer providing the foundation for the actual simulator functions—the kernel of the software system. The main components here are the Neuron Manager, responsible for the instantiation of neurons, and the Connection Manager, responsible for creating the synaptic connections. At the highest level, a C-API provides Create(..), Connect(..), and Simulate(..) function calls, which represent a minimal set of functions required to instantiate and simulate a network. Besides the simulator core-functionality, we implemented functions for system configuration, testing and debugging as well as for user-interactive node control. Access to those is given through a serial user console interface. To minimize the resources footprint and achieve best possible performance, the software system was implemented as bare-metal application, running natively without the use of any underlying operating system. When executed, it makes use of one of the two ARM Cortex-A9 cores that the APU provides. During the execution of a Simulate(..) function call, no operations on the external memory are performed by the APU. This allows the PL to make optimal use of the bandwidth of the external memory while a simulation is running.

#### 4.2.1. Node-Local Network Instantiation

The current HNC node prototype requires that the neural network model is formulated as a sequence of Create and Connect function calls, which needs to be compiled to an executable. In this object-format it is loaded into the external memory and executed when a Simulate function call is issued. Each Create instantiates a single neuron. The function takes as its arguments a model name, the initial values of the neuron's state variables, and a logical neuron-id, which identifies the neuron on the node. The Create function calls are processed by the Neuron Manager. It maps the logical neuron-id to a dedicated hardware resource identified by a resource-id, i.e., a processing unit and a position in the ODE pipeline. This process mainly consists of setting up the data structures for state variables in memory while administering byte-orders and data type conversions according to the model-specific hardware implementation. The DMA controller operates directly on these data structures when the processing units are “*loaded”* and the state variables are moved to the SVBs—and also vice versa when “*unloaded”* and the data is read back to external memory. In the current implementation, an interrupt-controlled DMA operation takes ≈30μ*s* to fill the SVBs while 16KiB of data is transferred in order to load or unload the states of *N*^P^ = 1024 neurons.

Analogous to the Create function call for the instantiation of a neuron, a Connect function call creates a single connection. It expects in its argument list a logical source neuron-id (for a multi-node system extended by a node-id), a logical target neuron-id, as well as the synapse parameters, i.e., a weight and a delay. From the sequence of Connect function calls, the Connection Manager builds the data structures in the external memory that represent the network connectivity. This structure associates each source neuron with a list of synapse target connections.

#### 4.2.2. Recording

The HNC node implements two different solutions for recording the network activity data, one for recording spikes and one for recording state variables. Recording spike events is a fully asynchronous process which is decoupled from the simulation scheduling. During a simulation, the spike events are grouped together as they occur and packed to 64-bit values which are buffered in the Recording FIFO (shown at the bottom in Figure 6) before being written to external memory. The high performance port HP3 is used for the write operations. Its read channel is already assigned to the retrieving of the presynaptic data. Sharing the port—and the external memory—does not create any visible read-write contention. Performance measurements carried out with and without spike recording did not show any degradation in performance with active spike recording and led to comparable results for the measured acceleration factors, even at high spike rates. The current design implements a recording buffer with a size of 60MiB capable of caching ~15M spike events. This buffer is written by the recording hardware in a round robin manner and emptied by the simulation kernel's Recording Client (Figure 7), which transfers the data *via* a TCP connection to a TCP server running on the Linux host system. For the client implementation on the HNC node the open-source *lightweight IP*^13^ (lwIP) TCP/IP stack was used, which comes with the Xilinx board support package, and is included in the Vivado SDK. In order to read out state variables, a running simulation must be halted to allow the DMA controller to access the SVBs. Consequently, capturing state variables significantly reduces performance. On the other hand, the DMA provides the APU with an efficient way to access all state variables at once and at any desired interval.

### 4.3. Microarchitecture

The module microarchitectures presented in this section try to bring the data and the operations performed on them as close together as possible. The implementations aim at optimal low-latency solutions utilizing SoC device features, such as low-latency BRAM and high-performance streaming interfaces for external memory access.

#### 4.3.1. Connectivity Representation and Presynaptic Data Distribution

**Figure 8:**
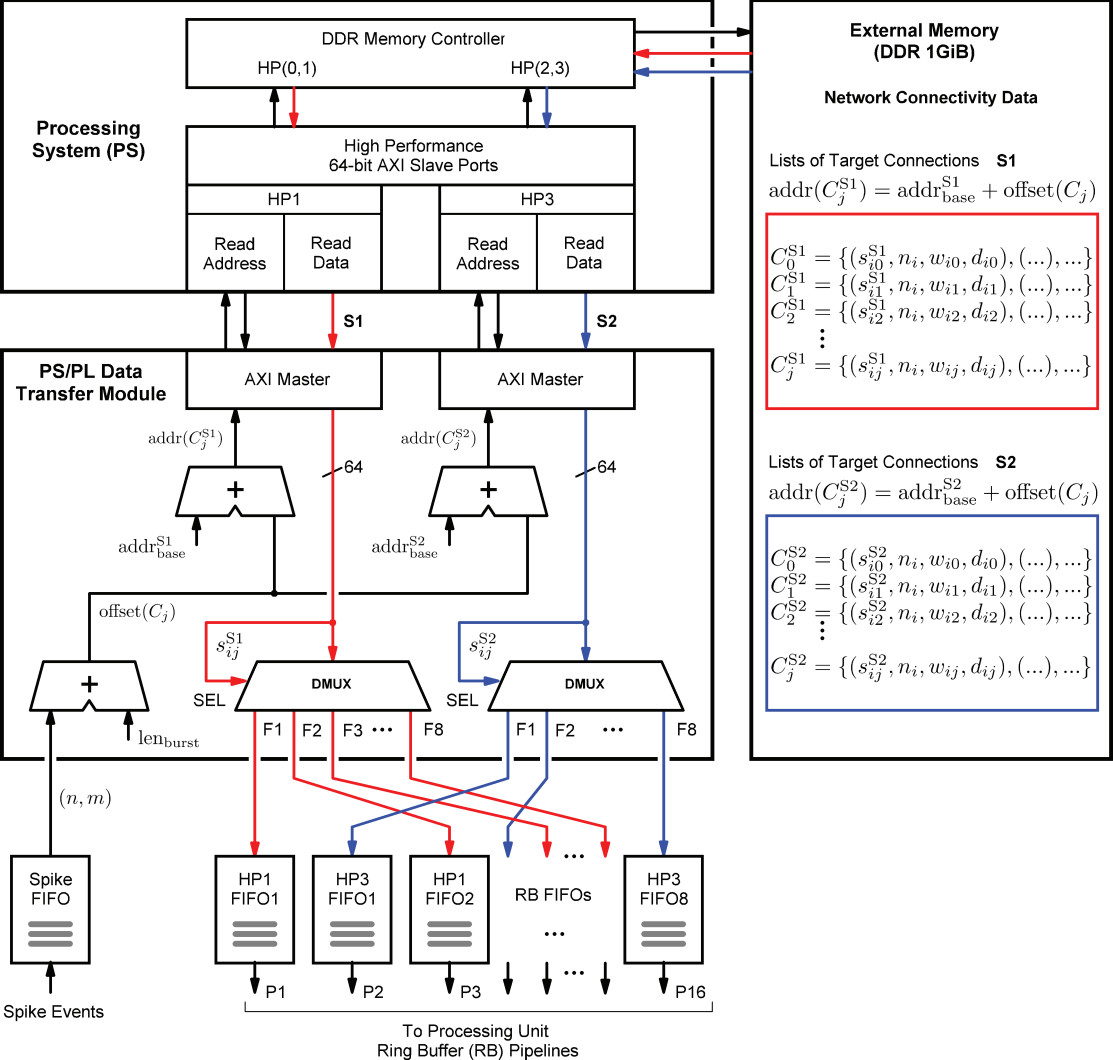
Presynaptic data distribution. Upon the arrival of a spike event, the presynaptic data is read from the external memory in two independent parallel data streams S1 and S2, indicated by the red and blue arrows, and distributed to the RB FIFOs by the demultiplexers (DMUX). While the *Processing System (PS)* performs the external storage operations bypassing the APU (not shown in the figure), the *PS/PL Data Transfer Module* controls the two AXI data streams and the high-performance ports HP1 and HP3 through which it connects to the PS. It calculates the memory addresses of two lists, $C_{j}^{\text{S}1}$ and $C_{j}^{\text{S}2}$ which constitute the two data streams, that is, the presynaptic data associated with the neuron that has emitted the spike. This data is stored in two different memory regions, marked by the red and blue boxes.

The structure in which the network connectivity data is stored in memory is determined by the microarchitecture of the *PS/PL Data Transfer Module*, which is shown in Figure 8. Upon the arrival of a spike event, it retrieves the list of synapse target connections *C*_*j*_ associated with a source neuron *n*_*j*_, and distributes the data items to the RB FIFO buffers for further processing by the RB pipelines (see also Figure 6). Such a retrieved list constitutes the *presynaptic data*. It is represented by a list of quadruples *C*_*j*_ = {(*s*_*ij*_, *n*_*i*_, *w*_*ij*_, *d*_*ij*_), .., ()}, where *n*_*i*_ specifies the target neuron, *w*_*ij*_ and *d*_*ij*_ denote the synaptic weight and delay values, and *s*_*ij*_ is a data path control value assigning a data item to its associated RB FIFO buffer by controlling the demultiplexer circuits (DMUX, Figure 8). The data format of the synaptic target list items is detailed in Supplementary Figure S1. The demultiplexers connect the data paths alternately with the RB FIFO buffers and thus the processing units. This architecture detail comes in handy when removing, adding, or combining processing units, as it helps to maintain a balanced load on the high-performance ports. The design and implementation of the module aim at lowest possible data access latency and an optimal utilization of the available read bandwidth of the external memory. Therefore, the *PS/PL Data Transfer Module*, residing in the PL, is interfaced with the PS, and thus with the external memory, through the two high-performance ports HP1 and HP3. This splits the target list into the two lists $C_{j}^{\text{S}1}$ and $C_{j}^{\text{S}2}$ assigned to HP1 and HP3, respectively. Their assignment (and associated data paths) are indicated in red and blue in Figure 8. The two high-performance ports are capable of working in parallel and independently of one another, while for example, the ports HP0 and HP1 would share the same PS resources, hindering full parallelism.

**Figure 9:**
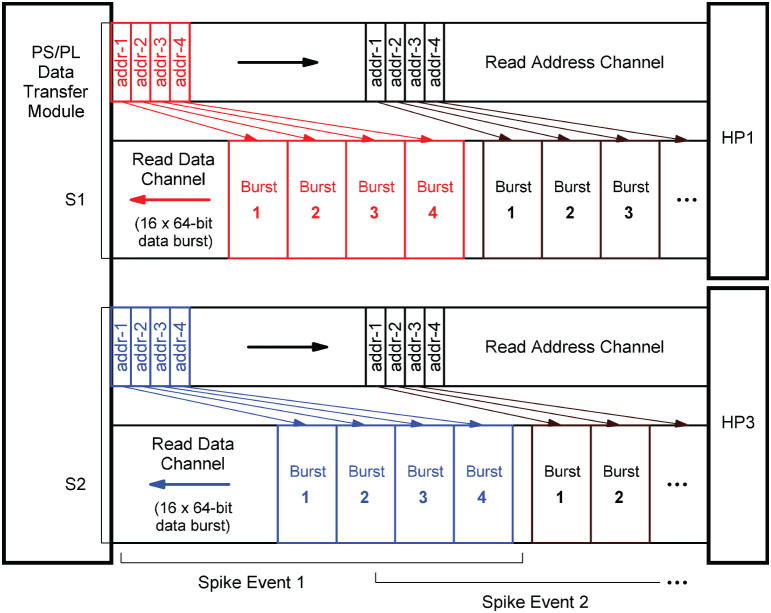
AXI stream protocol implementation. To create data streams that are as continuous as possible, data transfers are already scheduled without waiting for the preceding transfer to complete. Per spike event, the transfer of a sequence of four data bursts is initiated on each of the two read data channels associated with the two streams S1 and S2 (marked red and blue). For this purpose, the memory read base addresses of the four burst data packets are transmitted as a block on the read address channels.

In terms of implementation, the port interfaces follow the *Advanced eXtensible Interface*^14^ (AXI) standard (Arm Limited, 2021). More precisely, they provide 64-bit AXI3 Slave interfaces. On the PL, the *PS/PL Data Transfer Module* architecture bundles two AXI Master stream interface implementations that constitute their counterparts. The AXI protocol is based on data bursts. The presynaptic data to be retrieved upon the occurrence of a single spike event is transmitted in two parallel sequences of four bursts, i.e., four bursts on each port, where a burst consists of 16 64-bit data items. The principle is shown in Figure 9. The red and blue colors correspond to the datapath coloring in Figure 8. The read address channels describe the address and control information of the data bursts transferred on the read data channels. The addresses $\left(\text{addr}\left(C_{j}^{\text{S}1}\right),\text{addr}\left(C_{j}^{\text{S}2}\right)\right)$ are calculated from the neuron-id and node-id (*n*_*j*_, *m*) of the source neuron that emitted the spike, the burst length (len_burst_), and the memory base addresses of the two target lists $\left(\text{add}{\text{r}}_{\text{base}}^{\text{S}1},\text{add}{\text{r}}_{\text{base}}^{\text{S}2}\right)$.

In order to generate data streams that are as continuous as possible, read operations that are triggered by subsequent spike events are already scheduled even though the read data channels are still occupied. By this means, the two data streams S1 and S2 are created. Every spike event triggers a transfer of a 1KiB data packet from external memory. For a single data packet, an average transmission time of ~550 ns (*f*_clk_ = 200 MHz) was measured. This corresponds to a data transfer rate of 1818 MiB/s which is a much higher throughput than achievable with a Xilinx AXI DMA soft IP core (Xilinx, 2019a)—the common solution for high-bandwidth direct memory access. The DMA soft IP core throughput is specified with 399.04 MB/s at 100 MHz clock frequency (Xilinx, 2019a).

The transfer parameters, the number of bursts and the size of a burst, are configurable in control registers. They were set as discussed above allowing a source neuron to make 128 synapses on a node. In the current prototypical implementation, the transferred data packets are of same size for all spike events. Unused list entries are read from memory but they are not distributed.

The RB FIFO buffers which connect the *PS/PL Data Transfer Module* with the processing units serve two purposes. First, they buffer the synaptic input derived from incoming spike events for the time that the ODE pipelines are operating on the ring buffers (RBs) and blocking them for parallel read operations, and second, they allow a clock domain crossing. We have not yet investigated the latter, but it would allow the *PS/PL Data Transfer Module* to operate at a higher clock frequency than the processing units, which could have a positive impact on the latency of external memory data access.

#### 4.3.2. Ring Buffer Processing and Ordinary Differential Equation Solver Pipeline

**Figure 10:**
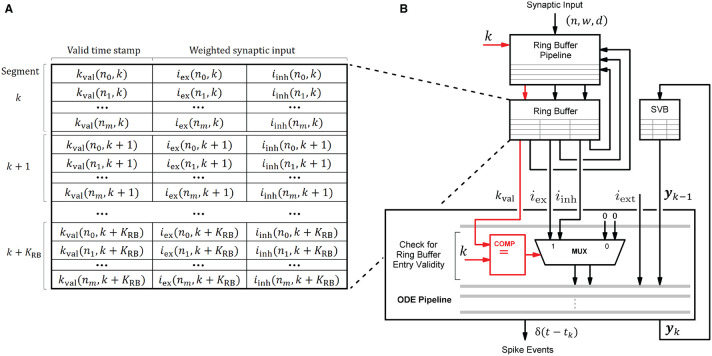
Ring buffer (RB) architecture and interaction of components. Local variables are stored in the ring buffer (RB) and the state variables buffer (SVB). For their implementation fast, low-latency on-chip BRAM memories were used. Shown is the interaction of the RB pipeline and the ODE pipeline which both operate on the RB. To avoid additional write operations by the ODE pipeline to invalidate the RB entries when processed, each entry is provided with a time stamp *k*_val_ that indicates the RB cycle for which it is valid. The value of *k*_val_ is calculated by the RB pipeline—see also the RB update algorithm (Figure 11)—and compared with the current simulation time step to verify an entry's validity when read by the ODE pipeline for processing. The principle is illustrated in **(B)** where the data path is marked in red. The corresponding RB layout is shown in **(A)**.

The HNC node's processing units draw their ability to accelerate computations primarily from the pipelined processing when accumulating the synaptic inputs in the ring buffers, and when progressing the neuron and synapse model dynamics in the ODE pipelines. This capacity builds on the usage of fast, low-latency on-chip BRAM for storing local variables. Figure 10 shows the involved components and their interaction for a single processing unit. In every simulation time step, the ODE pipeline updates the state vectors of 64 neurons (***y***_*k*_ → ***y***_*k*+1_) while operating on the state variables buffer (SVB). The SVB is implemented as true dual-port BRAM enabling high pipeline-throughput and minimal iteration latency. The state vectors are implemented as 128-bit data words, where 120 bits are available for use to store the state variables and 8 bits are required for pipeline control. The representation of the 120-bit data word, in terms of the number of state variables, their length and type, is determined by the model's hardware implementation and its counterpart in the software system, namely the function for neuron instantiation as part of the neuron manager. This generic approach allows a certain flexibility with regard to the choice of data types and operations according to the numerical precision required by the model to be implemented. This architecture is open to extensions, as the ODE pipeline module can be exchanged to support a wide variety of neuron and synapse models. An example implementation is given in Supplementary Figure S3. It shows the microarchitecture of the Izhikevich model implementation used for the performance evaluation and validation task conducted in this work.

An ODE pipeline retrieves the accumulated synaptic inputs *i*_ex_ and *i*_inh_ from the RB and may also receive input from an external source, such as a PRNG. Like the SVB, the RB is also implemented as true dual-port BRAM. The buffer layout, shown in Figure 10A, consists of *K*_RB_ segments subdivided into *N*^P^ = 64 entries - the number of neurons in the pipeline. The RB is read in a round-robin fashion by the ODE pipeline, such that a segment is re-addressed after *k* + *K*_RB_ simulation time steps. The delay resolution—the minimum of which is given by the simulation resolution, i.e., *d*_min_ = *h* = 0.1 ms—and the number of segments *K*_RB_ determine the maximum possible synaptic delay.

RB entries that have already been processed, and are thus outdated, remain in the buffer and may be erroneously reprocessed by the ODE pipeline in subsequent RB cycles. In order to avoid having to add an additional write operation to the ODE pipeline to mark an entry as processed, and thus invalid, we implemented a solution which turns this approach around. When updated, an entry is marked with a time stamp *k*_val_ that indicates the RB cycle for which the entry is valid. The principle is shown in Figure 10B. This *valid time stamp* is derived from the calculated target simulation step $k^{\prime }\leftarrow k+d_{ij}$ excluding the lower log_2_(*K*_RB_) digits. Upon entering the ODE pipeline, the higher order bits of *k* and the value of *k*_val_ are checked for equality. If this is the case, *i*_ex_ and *i*_inh_ are valid synaptic inputs. This method further avoids the restoring of RB entries in the situation of an ODE pipeline restart (see below). The disadvantage of this solution is a higher consumption of the scarce BRAM resources.

**Figure 11:**
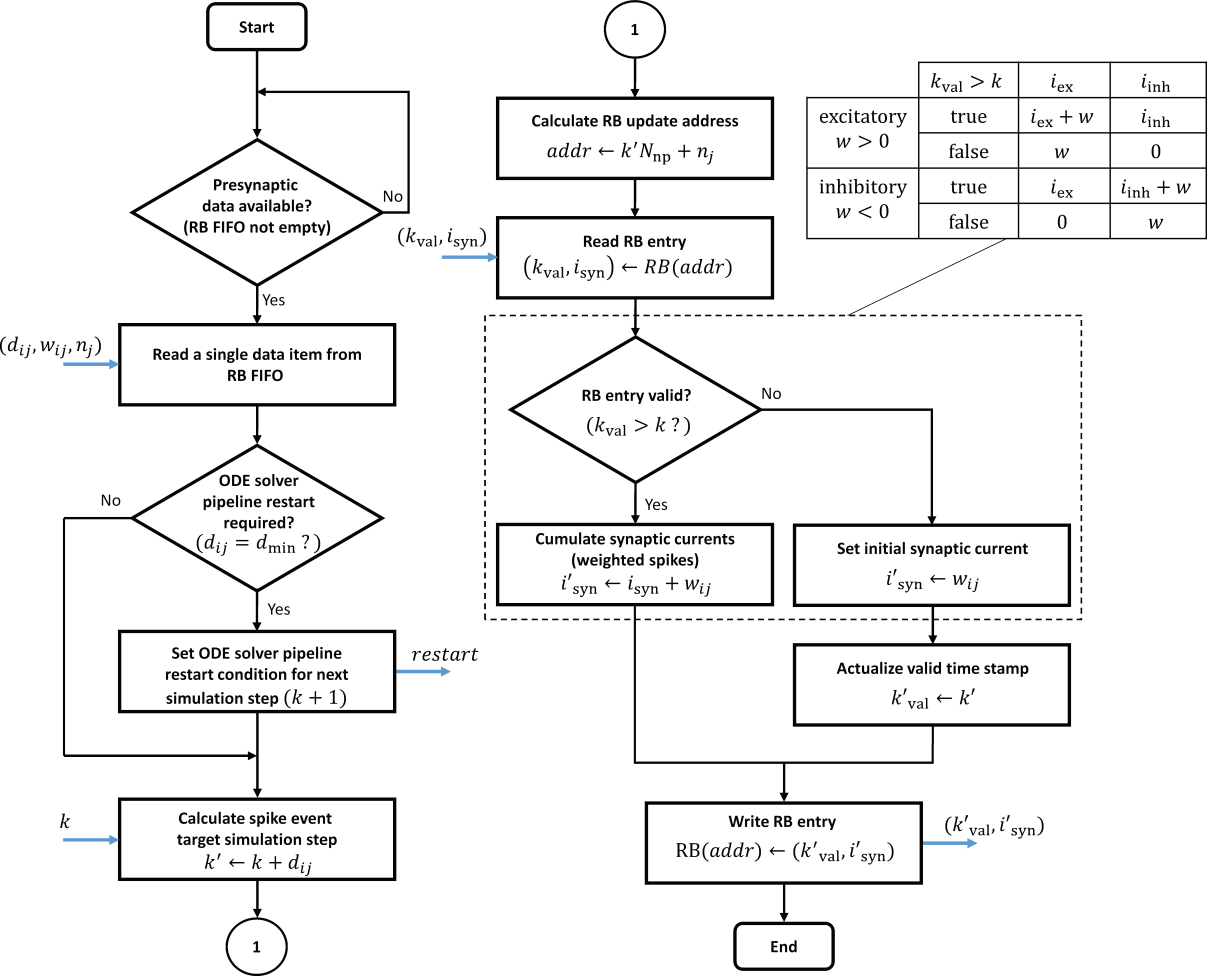
Ring buffer update algorithm. The algorithm is executed by the RB pipelines. The blue arrows indicate in- and out-going data items at different pipeline stages. The dashed box is for simplified illustration and shows the algorithm for the exceptional case of static synapses where a neuron's excitatory and inhibitory synaptic input can be lumped together. In all other cases, the algorithm expands according to the table on the upper right.

In contrast to the ODE pipelines that are controlled by a finite state machine, an RB pipeline works in a purely event-driven fashion. When not stalled by ODE pipeline operations, the presynaptic data buffered in the RB FIFO is being fetched. It is then passed through the RB pipeline which executes the ring buffer algorithm detailed in the flow diagram in Figure 11.

The proposed design raises two issues of potential read-before-write conflicts which need to be taken into consideration. Even though RB update operations never address an RB segment that is processed in the current time step *k*, it may nevertheless happen that an RB write operation is not considered in further processing. This can be the case if a presynaptic data item represents a synapse with *d*_*ij*_ = *d*_min_, i.e., a 0.1 ms delay. The initiated update on the *k* + 1 RB segment may have no effect as it is already being fetched into the ODE pipeline for the next simulation step. In such a case, the ODE pipelines must be reset and restarted. This adds an additional latency *L*_ODE_ to the processing, where *L*_ODE_ denotes the pipeline depth. In the proposed design the ODE pipeline restart is software controlled. Whether a restart condition is indicated or not depends on the synaptic delay value and is encoded in the presynaptic data (see table in Supplementary Figure S2). This information is passed to the finite state machine that is controlling the ODE pipeline operation and considered when the next simulation step is initiated. Another read-before-write conflict arises in the RB pipeline itself, caused by BRAM read, write, and operation latencies. These must be taken into account if consecutive presynaptic data items initiate updates on the same RB entry. The reading of an entry for which a previous write operation has not yet completed will lead to a wrong synaptic input value. This problem may only arise with multapses. It can also be solved in software by rearranging the lists of synaptic targets in memory.

It is also worth mentioning that a ring buffer shares its read ports between the RB and ODE pipelines. We have investigated the impact of read contention on performance due to concurrent read operations. When not considering an asynchronous external spike input and long ODE pipeline iteration latencies, only an early arriving spike event may find the RB pipeline stalled when placing data in the RB FIFOs. The additional latency is minimal (in the order of a few clock cycles per simulation time step) and thus can be neglected.

#### 4.3.3. Operating Latencies

**Figure 12:**
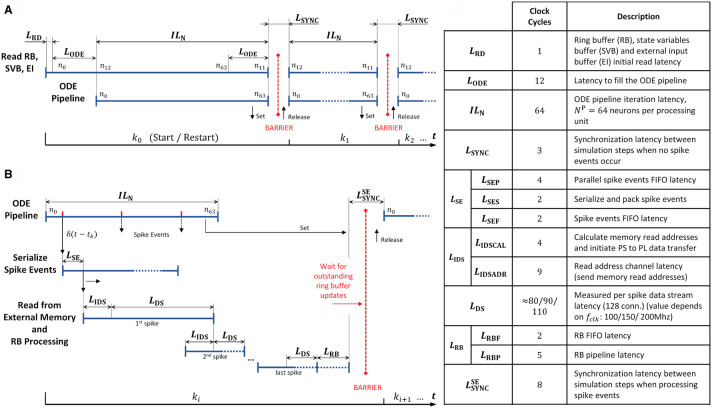
Operating latencies. The scheduling of operations and the latencies associated with it, distinguishes two basic cases. **(A)** If no spike events occur, operation mainly reduces to ODE pipeline processing. **(B)** Normally, spike events have to be processed which changes and adds latencies. Postsynaptic spike events must be serialized, and for incoming presynaptic spike events the presynaptic data must be retrieved from external memory. **(C)** Table listing relevant latencies. The value of *L*_DS_ cannot be derived from the microarchitecture; its average value was determined using an external logic analyzer.

In order to further examine the design, we extracted the operating latencies from the microarchitecture VHDL implementation, or where that was not possible, measured them with an external logic analyzer. The timing diagrams and the table in Figure 12 details the performance relevant operating latencies of the HNC node in clock cycles and show the timing of the operation scheduling.

At simulation start (and restart) the ODE pipelines are empty. An initial memory read operation that fetches the first data items, and the process of filling the ODE pipelines results in the latencies *L*_RD_ and *L*_ODE_. This is illustrated in Figure 12A for the case of two simulation steps in which no spike events occur. The latency *L*_ODE_ corresponds to the depth of the ODE pipelines and may differ depending on the implemented model. The same holds for the iteration latency *IL*_N_, which is the number of clock cycles required to process all *N*^P^ = 64 neurons assigned to a pipeline. At the end of a simulation step a few clock cycles *L*_SYNC_ are required for synchronization.

Spike events can occur in every clock cycle of the ODE pipeline operation, as depicted in Figure 12B. They are serialized and packed, resulting in a latency of *L*_SE_ (see also the table in Figure 12C). Before the presynaptic data can be read from external memory, its memory addresses have to be calculated. The latencies created by this process are summarized in *L*_IDS_. The high-performance ports and the memory controller on the PS, as well as the external memory itself, determine the overall read access latency, and hence the value of *L*_DS_ as the data is streamed into the RB FIFOs by the *PS/PL Data Transfer Module*. The components involved are connected to different clock domains and contribute with latencies that are determined by the SoC technology rather than by the implemented user logic. We therefore measured the value as the number of PL clock cycles required for the transfer of a 1KiB data packet—the amount of data which is read from external memory upon the occurrence of a single spike event—for three PL clock frequencies *f*_clk_ = 100/150/200 MHz.

At the end of a simulation step in which spike events had to be processed, the RB pipelines might still be filled, and pending RB updates must be finalized. This adds the latencies summarized in *L*_RB_. Finally, the HNC node goes into synchronization to prepare for the next simulation step. This requires a few clock cycles at the end of a simulation step compared to the situation where no spike event occurred. This adds the latency $L_{\text{SYNC}}^{\text{SE}}$ to the processing.

In a multi-node system, the total latency would be extended by inter-node synchronization times. This is not explicitly included in the timing diagrams in Figure 12 but indicated by the red barriers.

## 5. Methods and Materials

### 5.1. Workload Model

The synchronous, time-driven neuron states update process (red arrows in Figure 1) generates a computational cost determined almost exclusively by the number of neurons processed by a single processing unit, and thus adds a constant operating latency. In contrast, the computational cost of the asynchronous, event-driven process of presynaptic data distribution and processing (blue arrows in Figure 1) depends mainly on the amount of presynaptic data to be processed and retrieved from external memory. The amount of data is determined by the average number of synapses on a node that a source neuron connects to *C*^M^, as well as the total number of spike events processed by the node. For a given number of neurons per node *N*^M^—which is a hardware design parameter and a constant—a certain number of nodes *M* is required to simulate a network of size *N*. The connection probability ϵ of the network determines the average in/out-degree *K* = ϵ*N*, i.e., the number of in- and out-going synaptic connections of a neuron, which grows with the network size. Since the connections are distributed across the nodes, the average number of synapses on a node that a source neuron connects to remains constant for a given ϵ, even if the network size is growing. This is expressed by Equation (1).

(1) $$\begin{array}{r} C^{\text{M}}=\epsilon N^{\text{M}}=\frac{\epsilon N}{M} \end{array}$$

Because *C*^M^ is a constant, the average amount of presynaptic data retrieved from external memory is consequently of same size for every spike event. It is therefore practical to consider as an indicator of computational workload the average number of spike events processed per simulation time step *k*:

(2) $$\begin{array}{r} {\bar{\nu }}_{\text{k}}=N\bar{\nu }h,\;\text{with }\bar{\nu }=\frac{1}{N}\underset{\text{N}}{\sum }\frac{n_{\text{sp}}\left(T\right)}{T} \end{array}$$

(3) $$\begin{array}{r} {\bar{\nu }}_{\text{k}}=\frac{h}{T}\underset{\text{N}}{\sum }n_{\text{sp}}\left(T\right) \end{array}$$

where $\bar{\nu }$ is the average firing rate calculated over all neurons in the network, *n*_sp_ is a neuron's total spike count in the interval *T*, and *h* defines the temporal resolution, the step size, of the grid-based simulation, i.e., the time interval *h* = Δ*t* = *t*_*k*+1_ − *t*_*k*_. Note that this metric is initially independent of the number of neurons simulated.

### 5.2. Performance Model

We exploit knowledge of the HNC node microarchitecture latencies to derive a performance model that allows conclusions to be drawn about the performance characteristics in different scenarios regarding the workload and design and technology parameters. We make the following assumptions that represent a scenario that maximally challenges the hardware:

- *All neurons have at least one target connection with a synaptic delay value d*_*ij*_ = *d*_min_.
  Every spike event will initiate an ODE pipeline restart. This adds the latencies *L*_RD_ and *L*_ODE_ (Figure 12) to every simulation step.
- *Spike events are distributed uniformly across the neurons in an ODE pipeline and over pipeline iterations*.
  We assume that the expected value for the timing of a spike event is the middle of an ODE pipeline iteration, i.e., at *IL*_N_/2. This is justified by the two-population Izhikevich network used for the benchmarking (Sec. 5.3), and the placement of the neurons on the processing units.
- *All lists of synaptic target connections are the same length*.
  This is justified by the current design (Section 4.3.1). Upon every spike event, a 1KiB data packet is transferred from external memory to the RB FIFOs.

As explained in the previous section, we take the average number of spike events ${\bar{\nu }}_{\text{k}}$ processed in a single simulation step *k* as a measure of the workload. The time span to perform a single simulation step becomes minimal if no spike events occur, and is predominantly determined by the number of serially processed neurons assigned to an ODE pipeline. This is reflected in the ODE pipeline iteration latency *IL*_N_. Together with the synchronization latency *L*_SYNC_, it sets the upper bound for the single-node acceleration factor $F_{\text{S}}^{\text{MAX}}$ at a given clock frequency *f*_clk_. From the timing diagram in Figure 12A we derive:

(4) $$\begin{array}{r} F_{\text{S}}^{\text{MAX}}=\frac{khf_{\text{clk}}}{L_{\text{RD}}+L_{\text{ODE}}+k\left(IL_{\text{N}}+L_{\text{SYNC}}\right)} \end{array}$$

where *k* denotes the number of simulation steps, and *h* specifies the temporal resolution of the simulation, i.e., *h* = Δ*t* = 0.1 ms. For *k* ≫ 1 this simplifies to

(5) $$\begin{array}{r} F_{\text{S}}^{\text{MAX}}=\frac{hf_{\text{clk}}}{L_{\text{Σ}}} \end{array}$$

where *L*_Σ_ = *IL*_N_ + *L*_SYNC_. Analogously to *L*_Σ_, which summarizes the processing latencies for the non-spiking case, latencies arising from processing spiking events can be summarized according to the timing diagrams and process scheduling shown in Figures 12A,B. This consists of the sum of the latencies for the spike events serialization and buffering process (*L*_SE_ = *L*_SEP_ + *L*_SES_ + *L*_SEF_), the latencies incurred by the initiation of the data streams S1 and S2 (*L*_IDS_ = *L*_IDSCAL_ + *L*_IDSADR_), see Figure 9, and the latencies resulting from the processing of outstanding presynaptic data items at the end of a simulation step (*L*_RB_ = *L*_RBF_ + *L*_RBP_). The number of clock cycles for each latency, as well as its description, can be found in Figure 12C. Altogether, this results in

(6) $$\begin{array}{r} L_{\text{Σ}}^{\text{SE}}=L_{\text{RD}}+L_{\text{ODE}}+\frac{IL_{\text{N}}}{2}+L_{\text{SE}}+L_{\text{IDS}}+L_{\text{RB}}+L_{\text{SYNC}}^{\text{SE}} \end{array}$$

The term *IL*_N_/2 in Equation (6) reflects the assumption of a uniform distribution of the spike events.

For an isolated node with no inter-node communication, the acceleration factor as a function of the average number of spike events per simulation step can now be formulated as follows:


(7) $$F_{\text{S}}({\bar{\nu }}_{\text{k}})=\;\{\begin{array}{l} \frac{hf_{\text{clk}}}{{\bar{\nu }}_{\text{k}}(L_{\Sigma }^{\text{SE}}+L_{\text{DS}})+(1-{\bar{\nu }}_{\text{k}})L_{\Sigma }}\text{ if }{\bar{\nu }}_{\text{k}}<1\\ \frac{hf_{\text{clk}}}{L_{\Sigma }^{\text{SE}}+{\bar{\nu }}_{\text{k}}L_{\text{DS}}}\text{ otherwise} \end{array}\;$$

For ${\bar{\nu }}_{\text{k}}<1$, the denominator in Equation (7) consists of two terms corresponding to the spiking $\left[{\bar{\nu }}_{\text{k}}\left(L_{\text{Σ}}^{\text{SE}}+L_{\text{DS}}\right)\right]$ and the non-spiking $\left(\left(1-{\bar{\nu }}_{\text{k}}\right)L_{\text{Σ}}\right)$ case, where *L*_DS_ denotes the per spike event data stream latency. The two branches are equal for ${\bar{\nu }}_{\text{k}}=1$. In the absence of spike events, $F_{\text{S}}\left(0\right)=F_{\text{S}}^{\text{MAX}}$ applies (see Equation 5).

Please note that Equation (7) does not consider *C*^M^ - the average number of synapses on a node that a source neuron connects to. The value of *C*^M^ determines the value of *L*_DS_ that was measured since it cannot be derived from the microarchitecture. This becomes relevant, for example, when the number of neurons per node *N*^M^, and thus also *C*^M^ changes (Equation 1). Furthermore, it neglects the possibility that a presynaptic data transfer could complete before all neurons are processed, i.e., *IL*_N_/2 − *L*_DS_ > 0. To account for this, the value of $L_{\text{Σ}}^{\text{SE}}$ would have to be corrected by adding *IL*_N_/2 − *L*_DS_. However, one would only see an effect at very low spike rates because ${\bar{\nu }}_{\text{k}}L_{\text{DS}}\gg IL_{\text{N}}/2-L_{\text{DS}}$ applies. Equation (7), therefore, represents a good estimate of the acceleration factors that can be achieved with the proposed HNC node design under different workloads.

Currently, only a single-node prototype exists. In order to estimate the performance characteristics of a multi-node system, we expand the performance model to include inter-node communication latencies. Strongly simplifying the complex effects of communication network topologies, protocols, and low-latency interconnects, we propose three basic assumptions:

- Spike events are broadcasted, i.e, communicated to all nodes.
- Inter-node connections all have the same and fixed transmission latency time *T*_COM_, which adds to every simulation step. In addition to the times needed to communicate the spike events between nodes, *T*_COM_ also includes inter-node synchronization latencies, i.e., barrier messaging times.
- To take into account that inter-node communication increase with workload, every spike event adds a transmission latency to the communication, i.e., a variable, workload dependent portion defined as a small fraction of the transmission latency time. It is specified by a factor α and results for a given workload in ${\bar{\nu }}_{\text{k}}\alpha T_{\text{COM}}$.

Adding inter-node communication latencies to Equation (7) results in


(8) $$F_{\text{C}}({\bar{\nu }}_{\text{k}})=\{\begin{array}{l} \frac{hf_{clk}}{{\bar{\nu }}_{\text{k}}(L_{\Sigma }^{\text{SE}}+L_{\text{DS}}+\alpha L_{\text{COM}})+(1-{\bar{\nu }}_{\text{k}})L_{\Sigma }+L_{\text{COM}}}\text{ if }{\bar{\nu }}_{\text{k}}<1\\ \frac{hf_{clk}}{L_{\Sigma }^{\text{SE}}+{\bar{\nu }}_{\text{k}}(L_{\text{DS}}+\alpha L_{\text{COM}})+L_{\text{COM}}}\text{ otherwise} \end{array}$$

where *L*_COM_ denotes the transmission latency in PL clock cycles derived from the transmission latency time, i.e., *L*_COM_ = *f*_clk_*T*_COM_. Note that even in the absence of spike events, *L*_COM_ does not vanish as it includes inter-node synchronization times. According to Equation (4), the upper bound for the acceleration factor with inter-node communication then becomes:


(9) $$\begin{array}{r} F_{\text{C}}^{\text{MAX}}=\frac{hf_{\text{clk}}}{L_{\text{Σ}}+L_{\text{COM}}} \end{array}$$

From the performance characteristics derived above, the total relative performance loss *P*_TOT_ with respect to the maximum achievable acceleration can be estimated for different workloads as follows:

(10) $$\begin{array}{r} P_{\text{TOT}}\left({\bar{\nu }}_{\text{k}}\right)=P_{\text{S}}+P_{\text{C}}=\left(1-\frac{F_{\text{C}}\left({\bar{\nu }}_{\text{k}}\right)}{F_{\text{S}}^{\text{MAX}}}\right)\cdot 100\%. \end{array}$$

The total performance loss can be further subdivided into the losses caused by the HNC node-local spike processing (which mainly consists of retrieving and distributing the presynaptic data)

(11) $$\begin{array}{r} P_{\text{S}}\left({\bar{\nu }}_{\text{k}}\right)=\left(1-\frac{F_{\text{S}}\left({\bar{\nu }}_{\text{k}}\right)}{F_{\text{S}}^{\text{MAX}}}\right)\cdot 100\% \end{array}$$

and the loss caused by the inter-node communication


(12) $$\begin{array}{r} P_{\text{C}}\left({\bar{\nu }}_{\text{k}}\right)=\frac{F_{\text{S}}\left({\bar{\nu }}_{\text{k}}\right)-F_{\text{C}}\left({\bar{\nu }}_{\text{k}}\right)}{F_{\text{S}}^{\text{MAX}}}\cdot 100\%. \end{array}$$

### 5.3. Verification, Validation, and Benchmarking Model: Two-Population Izhikevich Network

We use a simple two-population model as the basis for both the performance measurements and the verification and validation of the correctness of the HNC node hardware and software implementation. The network consists of 1,000 Izhikevich-type neurons (Izhikevich, 2003), which follow the dynamics

(13) $$\begin{array}{r} \frac{dv}{dt}=0.04v^{2}+5v+140-u+i_{\text{syn}}\left(t\right)+i_{\text{ext}}\left(t\right),\;\text{with }i_{\text{syn}}\left(t\right)=i_{\text{ex}}+i_{\text{inh}} \end{array}$$

(14) $$\begin{array}{r} \frac{du}{dt}=a\left(bv-u\right) \end{array}$$

(15) $$\begin{array}{r} if\;v\geq 30\text{mV},\text{then}\{\begin{array}{l} v\leftarrow c\\ u\leftarrow u+d \end{array} \end{array}$$

The network consists of 800 excitatory regular spiking neurons [(*a, b, c, d*) = (0.02, 0.2, −65.0, 8.0)] and 200 inhibitory fast spiking neurons [(*a, b, c, d*) = (0.1, 0.2, −65.0, 2.0)]. The excitatory population makes random connections to the inhibitory population and to itself. The inhibitory population only projects to the excitatory population. All neurons in the network draw their connections with a fixed in-degree of *K*_in_ = 100 and receive additional input from an external source. A detailed description of the network is given in the Supplementary Material.

The choice of this model was motivated by our previous work, where we subjected a two-population Izhikevich network implementation on the SpiNNaker system to a rigorous verification and validation task (Gutzen et al., 2018; Trensch et al., 2018).

## 6. Discussion

We presented an SoC-based hybrid software-hardware architecture of a neuromorphic computing node. This is to be seen as a complementary yet distinct approach to the neuromorphic developments aiming at brain-inspired and highly efficient novel computer architectures for solving real-world tasks. The requirements for achieving reproducible hyper-real-time neuroscience simulations are different, so also the technical challenges. We examined the extent to which the proposed architecture and Xilinx Zynq SoC device technology is capable of meeting the high demands of modeling and simulation in neuroscience in terms of flexibility, accuracy, and simulation performance.

### 6.1. Flexibility

The HNC node design exploits the trade-off between flexibility and efficiency offered by the Xilinx Zynq SoC device technology. The tight coupling of programmable logic with a general purpose processor gives the developer the flexibility to cope with rapid developments in neuroscience and changing requirements. For example, the plethora of neuron and synapse models require that the operations and their scheduling performed by the ODE pipelines can be adapted in terms of the implemented numerical algorithms and data types. The application of code generation techniques (Blundell et al., 2018a) can abstract hardware implementation details away from a neuron and synapse modeling task. Therefore, the ODE pipeline architecture was implemented as a replaceable VHDL-module having a defined port interface. This makes the neuron and synapse model hardware implementations accessible to tools, such as NESTML (Plotnikov et al., 2016). By this means a wide variety of neuron and synapse models can be supported.

The availability of powerful, node-local processor cores also allows us to decentralize; moving tasks onto the neuromorphic compute nodes that are typically carried out on a host system. For example, the generation of the network connectivity could be carried out on a conventional system using established tools, such as PyNN (Davison et al., 2009) or PyNEST (Eppler et al., 2009), while the network instantiation process is parallelized by being delegated to the processor cores of the neuromorphic compute nodes. This would reduce network building times, especially when repeated simulations are performed (e.g., parameter scans). Moreover, the integration with the existing workflows for neural network modeling and simulation becomes easier to reach.

The HNC node architecture is open for extension, for example, the implementation of synaptic plasticity rules. Although plasticity models were deliberately left out for the current HNC node prototype, it was considered in the design decisions. In future developments, we intend to exploit the hybrid software-hardware architecture concept of the HNC node in such a way that plasticity algorithms programmed in software run on a dedicated plasticity processor—executed on the APU using the second, so far unused, ARM processor core—supported by accelerators implemented in programmable logic. To enable the implementation of spike-based plasticity rules (Morrison et al., 2008), the network connectivity data as well as the recorded spike events are stored in the external memory, thus keeping synaptic weights adjustable and spike history accessible to the processor cores. There are a number of different forms of plasticity (Magee and Grienberger, 2020) and a rapid development in the field which entails some technical challenges. The HNC node provides here a flexible platform as a means to explore novel architecture concepts to implement plasticity algorithms.

### 6.2. Numerical Precision

Particular care must be taken with respect to mathematical operations. Both the choice of data types and algorithms as well as their technical implementation require special attention. The design decisions made regarding the example Izhikevich neuron model ODE pipeline implementation (see Section 4 in the Supplementary Material), e.g., the data types and the numeric integration scheme, are based on the results of our earlier studies (Gutzen et al., 2018; Trensch et al., 2018). By conducting a calculation verification task^15^, we concluded that a 32-bit signed fixed-point data type (s16.15) does not provide the necessary numerical precision to capture the dynamics of the Izhikevich neuron model (Izhikevich, 2003) with sufficient accuracy. For the processing unit's ODE pipelines, we therefore implemented a 40-bit signed fixed-point data type (s16.23)—a decision also made to avoid expensive floating point operations. In combination with an explicit Forward Euler ODE solver method and an integration step size of *h* = 0.1 ms, we achieve sufficient accuracy—even though it is the simplest numerical method available. Analogously to the calculation verification task carried out in the studies mentioned, we verified the ODE pipeline operation by comparing the subthreshold dynamics and the spike timing to the results of an explicit Runge-Kutta-Fehlberg(4, 5) method with an absolute integration error of 10^−6^.

### 6.3. Verification of Implementations

During implementation, hardware and software components cannot be considered independently of each other and must therefore be developed in parallel in a co-development process. The HNC node software system is written in C and almost all hardware components were developed in VHDL. In contrast to a high-level synthesis approach, where a hardware design is formulated at an algorithmic level in the C language, for example, and the synthesis tool chain generates a reliable hardware description from it, the implementation in VHDL at the RTL level is rather error-prone. A well thought-out test strategy is therefore essential. It must consider the verification of the correctness of the technical implementation of the hardware and software components as well as the validation of the outcome of the simulations performed on the HNC node.

Our approach was that of an embedded hardware-software co-verification, in the sense of a directed software-controlled functional testing. For this purpose, the hardware components under test were connected to the APU of the SoC device through memory-mapped AXI-interfaces and subjected to a series of hierarchical functional tests written in C. These tests range from simple to complex and are executed on the APU. They include basic hardware and software functional tests, integration tests, as well as complex functional tests that also became part of the HNC node software system. Examples of such complex tests are memory read-write pattern tests. They ensure the correct implementation and operation of the DMA data transfer to and from the SVBs and verify data type and endianness conversion. Another example of a complex test scenario is the functional verification of the RB pipeline and RB buffer operation, where a software-controlled spike injection and a subsequent RB read out is used to verify the correctness of the presynaptic data processing.

### 6.4. Performance

Software developers of spiking neural network simulation tools invest much effort in the optimization of their codes to achieve best possible performance and simulation efficiency. They are well aware of the performance-critical nature of retrieving the presynaptic data from memory and its distribution, its accumulation in the ring buffers, and the update process of the neuron and synapse model dynamics performed at every simulation time step. The challenges in finding optimal solutions and implementations are manifold. For example, in large-scale networks, synaptic processing substantially dominates the computational load, and the irregular, random access pattern in retrieving the presynaptic data reduce a processor's cache hit rate and increases data access latencies (see e.g., Kunkel et al., 2014). The tools of trade here are algorithms that implement high parallelism in computations, “cache-friendly” data structures, and the application of techniques for latency hiding, such as data prefetching (Pronold et al., 2022). The proposed HNC node design aims to address these problems—which on conventional computer architectures are a consequence of the von Neumann bottleneck—by implementing performance-critical tasks in hardware. Specifically, the process of neuron and synapse model update benefits from the data-locality of state variables stored in fast on-chip BRAM memories. Storing the network connectivity data in an external memory, however, undermines this concept, and toward higher workloads, performance will be bound by external memory access latency. For larger systems and higher workloads, it is therefore crucial to aim for an architecture design that also allows data-locality for the presynaptic data processing. The design of the HNC node is constrained in this respect by the limited BRAM resources.

The ability to model the performance behavior for different design parameters is of great value as it can guide future developments and design decisions. We developed such a model for the HNC node architecture. The implementation strategy, based on the hardware description on register-transfer level (RTL) in the VHDL language has allowed us to derive an accurate performance model from the implemented microarchitecture. To this end we made several simplifying assumptions, in particular, with respect to the inter-node communication latencies. Network technologies are typically optimized for throughput, but not for latency. The value of the transmission latency time (*T*_COM_ = 500 ns) assumed for the performance evaluation is already ambitious. However, low-latency inter-node communication is as important for performance as data-locality is for the computations. Despite these simplifications, the model achieves a good approximation of the performance characteristics. Extrapolating from the single node performance, we predict that small clusters capable of simulating in hyper-real-time networks comprising a few tens of thousands of neurons would achieve acceleration factors in the order of 10 to 50.

### 6.5. Cluster Operation

Although cluster operation is not the focus of this article, some related considerations that influenced design decisions are worth mentioning. Three communication bottlenecks can be identified in the simulation flow that are relevant to the overall performance of a cluster system: the spike exchange between nodes, inter-node synchronization, and external communication for system configuration and operation including the unload of recorded simulation data. The requirements of these tasks differ in regard to latency and bandwidth. Inter-node spike communication and node synchronization require an ultra-low latency interconnect but not high bandwidth. The demand for external communication is completely different. For loading and unloading larger amounts of data, high bandwidth is desirable to achieve low system setup times and eventually real-time recording capability. We are therefore aiming at three different solutions tailored to the respective task, although our cluster concept is not yet fully developed. The HNC node encodes spike events using an *Address Event Representation* (AER; Mahowald, 1992). AER-based communication is well established in neuromorphic computing and the basis for low-latency spike-communication. In order to achieve the predicted cluster performance (cf. Section 3.2), it is crucial that the transmission latency time of *T*_COM_ = 500 ns for inter-node spike communication assumed by the performance model can be attained in a cluster consisting of a few tens of HNC nodes. The Xilinx Zynq SoC device used for the implementation of the HNC node prototype provides various hardware interfaces that would allow us to establish an efficient chip-to-chip communication, for example, a number of serial gigabit transceivers (GTX/GTH), PCI Express, and low-voltage differential signaling (LVDS) user I/Os (Xilinx, 2021). A solution for a low-latency spike communication in a 64-node FPGA cluster is, for example, presented in Moore et al. (2012). It exploits high-speed serial links and achieves a hop-latency of 50 ns in a 3D torus topology. For inter-node synchronization, we favor a simple one-wire (e.g., wired-or) solution where a global barrier signal is derived from the intra-node synchronization logic (cf. Figure 6). External communication with the HNC node is established using a 10/100/1000 Mb/s tri-speed Ethernet PHY and the TCP protocol—currently used only to stream the recorded simulation data to a host system. For a cluster, we aim at a *parallel data move* solution, in which each HNC node is connected to its own host system or host node, respectively.

The proposed technology and architecture is an ideal basis for prototyping and design space exploration—the primary domain of programmable logic devices—and for elaborating novel architectures. The reconfigurable logic allows extensive freedom in the implementation of the numerical models while the processor cores opens an elegant way to achieve system integration. They can be an intermediate step toward next-generation neuromorphic systems and neuroscience simulation platforms. In this sense, the proposed HNC node design complements the existing neuromorphic system architecture approaches of SpiNNaker and BrainScales, in regards both to technology and the trade-off between flexibility and efficiency.

## Data Availability Statement

The simulation scripts and source codes used in this work to demonstrate correctness are available online at: https://github.com/gtrensch/RigorousNeuralNetworkSimulations (10.5281/zenodo.6591552).

## Author Contributions

GT developed the System-on-Chip based hybrid architecture and implemented the prototype, developed the workload and performance model, and performed the experiments. GT and AM designed the experiments and wrote the paper. All authors contributed to the article and approved the submitted version.

## Funding

This project has received funding from the Helmholtz Association's Initiative and Networking Fund under project number SO-092 (Advanced Computing Architectures, ACA). Open access publication funded by the Deutsche Forschungsgemeinschaft (DFG, German Research Foundation)—491111487.

## Conflict of Interest

The authors declare that the research was conducted in the absence of any commercial or financial relationships that could be construed as a potential conflict of interest.

## Publisher's Note

All claims expressed in this article are solely those of the authors and do not necessarily represent those of their affiliated organizations, or those of the publisher, the editors and the reviewers. Any product that may be evaluated in this article, or claim that may be made by its manufacturer, is not guaranteed or endorsed by the publisher.

## Footnotes

^1^RRID:SCR_003833; http://neuralensemble.org/elephant.

^2^RRID:SCR_016543; https://github.com/INM-6/NetworkUnit.

^3^General Purpose Processor.

^4^Field Programmable Gate Array.

^5^Application-Specific Integrated Circuit.

^6^Maxeler Technologies: www.maxeler.com.

^7^A C-implementation of the network model is provided on GitHub: https://github.com/gtrensch/RigorousNeuralNetworkSimulations.

^8^A value of ${\bar{\nu }}_{\text{k}}=23.24$ spikes per simulation time step was determined experimentally from a 15 minutes NEST simulation using the implementation by van Albada et al. (2018).

^9^ https://github.com/gtrensch/RigorousNeuralNetworkSimulations

^10^JTAG is an industry standard named after the Joint Test Action Group.

^11^Universal Asynchronous Receiver Transmitter.

^12^Message Passing Interface, https://www.mpi-forum.org/.

^13^ http://savannah.nongnu.org/projects/lwip/

^14^The Advanced eXtensible Interface (AXI) standard is an extension of the Advanced Microcontroller Bus Architecture (AMBA), which is an open standard.

^15^Calculation verification tasks assess the level of error that arises from various sources of error in numerical simulations as well as to identify and remove them (Thacker et al., 2004).
