# Supplementary material for: A System-on-Chip Based Hybrid Neuromorphic Compute Node Architecture for Reproducible Hyper-Real-Time Simulations of Spiking Neural Networks
Source: Front Neuroinform. 2022 Jun 29;16:884033. doi: 10.3389/fninf.2022.884033 (PMC9277345; doi:10.3389/fninf.2022.884033)
Supplement: Supplementary file 1 [file Data_Sheet_1.pdf]

Supplementary Material: A System-on-Chip Based Hybrid Neuromorphic Compute Node Architecture for Reproducible Hyper-Real-Time Simulations of Spiking Neural Networks

1 PRESYNAPTIC DATA

The synaptic connections of a presynaptic neuron n_j to its postsynaptic targets $\{n_0, n_1, .., n_i\}$ (Fig. S1) are represented by a list of quadruples $C_j = \{(s_{ij}, n_i, w_{ij}, d_{ij}), .., ()\}$, where n_i specifies the target neuron, w_{ij} and d_{ij} denote the synaptic weight and delay values, and s_{ij} is a hardware control value. Such a list is retrieved from external memory upon the arrival of a spike event and forms the presynaptic data. The HNC node implementation encodes a single target list item in a 64-bit data format detailed in the table in Fig. S2. In addition to the synaptic weight and delay values, a data item also carries hardware control information stored in a 4-bit value. For example, if s_{ij} is set to $b'1111'$, the data is broadcasted to all processing units. This feature allows to efficiently perform a software-controlled reset of all ring buffers as well as to enable ring buffer testing by means of read-write BRAM memory pattern tests.

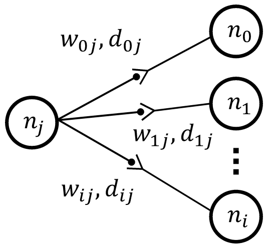

Figure S1. Synaptic target connections.

2 STATISTICAL MEASURES

To characterize the global dynamics of network activity, the following measures were calculated from the recorded spike trains.

Average firing rate: The number of spikes n_{sp} that a neuron emits in the interval T gives the average firing rate

$$FR = \frac{n_{sp}}{T} \qquad \text{(S1)}$$

measured in spks/s. Its distribution over the neurons is a measure to characterize the level of network activity.

Coefficient of variation: The measure analyzes the relative variability of the inter-spike intervals ISI_i calculated from the odered spike times t_i.

$$CV = \frac{\sqrt{\frac{1}{n-1}\sum_{i=1}^{n}(ISI_i - \overline{ISI})^2}}{\overline{ISI}}, \quad ISI_i = t_{i+1} - t_i, \quad \overline{ISI} = \frac{1}{n}\sum_{i=1}^{n} ISI_i \qquad \text{(S2)}$$

<table>
<tr><th>Bits</th><th>Data Type</th><th colspan="4">Description</th></tr>
<tr><td>39:00</td><td>40-bit signed fixed-point s16.23</td><td colspan="4">Synaptic weight, w_{ij}</td></tr>
<tr><td>48:40</td><td>up to 9-bit unsigned integer</td><td colspan="4">Synaptic delay, d_{ij}, in steps of 0.1 ms</td></tr>
<tr><td>54:49</td><td>6-bit unsigned integer</td><td colspan="4">Target neuron index within a processing unit, n $= \{0, 1, .., N^{\mathrm{P}} - 1\}$, $N^{\mathrm{P}}_{\mathrm{max}} = 64$</td></tr>
<tr><td rowspan="4">59:55</td><td rowspan="4">boolean</td><td colspan="4">Control flags</td></tr>
<tr><td>Bit</td><td colspan="3">Description</td></tr>
<tr><td>56</td><td colspan="3">Reset buffer segment</td></tr>
<tr><td>57</td><td colspan="3">Set if $d_{ij} = d_{\mathrm{min}}$ (restart ODE solver pipeline flag)</td></tr>
<tr><td rowspan="9">63:60</td><td rowspan="9">4-bit binary</td><td colspan="4">Encodes the ring buffer FIFO index from which DMUX SEL is derived, $s^{\mathrm{S1}}_{ij}, s^{\mathrm{S2}}_{ij}$</td></tr>
<tr><td rowspan="2">Value</td><td rowspan="2">Selected FIFOs</td><td colspan="2">Processing Units</td></tr>
<tr><td>Assigned to HP1</td><td>Assigned to HP3</td></tr>
<tr><td>b’0000’</td><td>none</td><td>none</td><td>none</td></tr>
<tr><td>b’1111’</td><td>F1, F2, F3, F4, F5, F6, F7, F8</td><td>P1, P3, P5, P7, P9, P11, P13, P15</td><td>P2, P4, P6, P8, P10 , P12, P14, P16</td></tr>
<tr><td>b’0001’</td><td>F1</td><td>P1</td><td>P2</td></tr>
<tr><td>b’0010’</td><td>F2</td><td>P3</td><td>P4</td></tr>
<tr><td colspan="4">…</td></tr>
<tr><td>b’1000’</td><td>F8</td><td>P15</td><td>P16</td></tr>
</table>

Figure S2. Synaptic target list item data format.

In (S2) n denote the number of neurons, and $\overline{ISI}$ defines the mean ISI_i (Shinomoto et al., 2003).

Pearson’s correlation coefficient: The measure quantifies the temporal correlation between all spike trains. It is determined by calculating the $n \times n$ matrix of the pairwise Pearson’s correlation coefficient between all combinations of n binned spike trains.

$$C[i, j] = \frac{< b_i - \mu_i, b_j - \mu_j >}{\sqrt{< b_i - \mu_i, b_i - \mu_i > \cdot < b_i - \mu_i, b_j - \mu_j >}} \tag{S3}$$

In (S3), $< .., .. >$ denotes the scalar product of two vectors, where b_i and b_j are the binned spike trains, and μ_i and μ_j represent their respective means (Grün and Rotter, 2010).

3 TWO-POPULATION IZHIKEVICH NETWORK MODEL

The following describes the neural network model used to examine the performance characteristics of the HNC node architecture, as well as to verify and validate the correctness of the HNC node software and hardware implementation.

The Izhikevich neuron model was originally published in Izhikevich (2003) and the two-population network model was published in Izhikevich (2006). The reproducibility of the latter study was evaluated in Pauli et al. (2018) using the NEST simulator, and in Trensch et al. (2018) and Gutzen et al. (2018) using the SpiNNaker neuromorphic system. The description and illustration in Tab. S1, S2 follow the proposed methods described in Nordlie et al. (2009) and Senk et al. (2021).

NETWORK DESCRIPTION

**Summary**	
**Populations**	excitatory population, inhibitory population
**Connectivity**	random, independent with fixed in-degrees, respecting Dale's principle
**Neuron model**	Izhikevich neuron model, regular spiking and fast spiking neuron model type
**Synapse model**	plastic synaptic weights (static for reproduction of network state), fixed delays
**Input**	random input

**Populations**		
**Name**	**Elements**	**Size**
E	Izhikevich, regular-spiking	$N_{\mathrm{E}} = \beta N$
I	Izhikevich, fast-spiking	$N_{\mathrm{I}} = N - N_{\mathrm{E}} = N - \beta N$

**Connectivity**		
**Source**	**Target**	**Pattern**
$E \cup I$	E	random, independent, fixed in-degree $K_{\mathrm{in}} = \epsilon N$, autapses and multapses prohibited ($\not A$, $\not M$), excitatory connection weight w_{E}, distributed excitatory connection delay $d_{\mathrm{E}} \sim \mathcal{D}$, constant inhibitory connection weight $\bar{w}_{\mathrm{I}}$, constant inhibitory connection delay $\bar{d}_{\mathrm{I}}$
E	I	random, independent, fixed in-degree $K_{\mathrm{in}} = \epsilon N$, multapses prohibited ($\not M$), constant inhibitory connection weight $\bar{w}_{\mathrm{I}}$, constant inhibitory connection delay $\bar{d}_{\mathrm{I}}$
E_{ext}	$E \cup I$	one-to-one δ, external input, I_{ext}

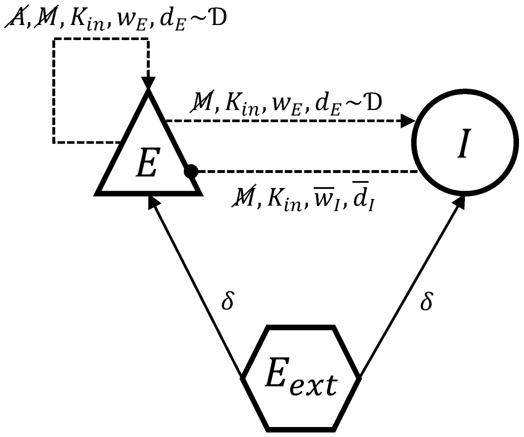

**Neuron**	
**Type**	Izhikevich model
**Description**	• dynamics of membrane potential $v_i(t)$ $(i \in \{1, \ldots, N\})$<br>$\frac{dv_i}{dt} = 0.04v_i^2 + 5v_i + 140 - u_i + I_i(t)$<br>$\frac{du_i}{dt} = a(bv_i - u_i)$<br>$\text{if } v_i \geq \theta, \text{then} \begin{cases} v_i \leftarrow c \\ u_i \leftarrow u_i + d \end{cases}$<br>• spike emission at t_k^i if $v_i(t_k^i) \geq \theta$<br>• initial values: $v_i(t=0) = V_0$, $u_i(t=0) = U_0$

Plasticity	
**Type**	additive spike-time-dependent plasticity (STDP) rule
**Description**	excitatory connections are plastic according to the STDP rule: $$w_\mathrm{E} \leftarrow \begin{cases} w_\mathrm{E} + A_+ \cdot \exp(\frac{-\Delta t}{\tau_+}) & : \Delta t \geq 0 \\ w_\mathrm{E} - A_- \cdot \exp(\frac{\Delta t}{\tau_-}) & : \Delta t < 0 \end{cases}$$ • update rule: synaptic weight changes are buffered for one biological second and then the weight matrix is updated for all plastic synapses simultaneously

Input	
**Type**	input of a constant current into a single neuron
**Description**	input to the network is a constant current I_ext into a single neuron $n \in (E \cup I)$ randomly selected in the interval Δt_ext

Table S1. Network description.

NETWORK PARAMETERS

Connectivity		
**Name**	**Value**	**Description**
N	1000	total number of neurons ($N_\mathrm{E} + N_\mathrm{I}$)
β	0.8	relative size of excitatory population
ϵ	0.1	connection probability
N_E	$\beta N = 800$	number of excitatory neurons
N_I	$N - N_\mathrm{E} = 200$	number of inhibitory neurons
K_in	$\epsilon N = 100$	number of synapses per neuron

Neuron		
**Name**	**Value**	**Description**
(a, b, c, d)	$(0.02, 0.2, -65.0, 8.0)$	model parameters: regular-spiking neuron type
(a, b, c, d)	$(0.1, 0.2, -65.0, 2.0)$	model parameters: fast-spiking neuron type
V_0	$-65.0\,\mathrm{mV}$	initial membrane potential $v_i(t=0)$
U_0	$0.2V_0 = -13.0\,\mathrm{mV}$	initial value of recovery variable $u_i(t=0)$
θ	$30\,\mathrm{mV}$	spike threshold

Synapse		
**Name**	**Value**	**Description**
w_E	6.0	initial excitatory synaptic weight (plastic)
w_I	-5.0	inhibitory synaptic weight
d_E	$[1, 2, ..., 20]$ ms	excitatory synaptic transmission delay, drawn from a uniform integer distribution
d_I	$1\,\mathrm{ms}$	inhibitory synaptic transmission delay

Plasticity		
**Name**	**Value**	**Description**
τ_+	20 ms	time constant, potentiation
τ_-	20 ms	time constant, depression
A_+	0.1 mV	amplitude, potentiation
A_-	0.12 mV	amplitude, depression
**Input**		
**Name**	**Value**	**Description**
I_{ext}	20 pA	external input current
Δt_{ext}	1 ms	external input current interval
**Simulation**		
**Name**	**Value**	**Description**
Δt	0.1 ms	time resolution

Table S2. Network parameters.

4 ODE PIPELINE EXAMPLE IMPLEMENTATION: IZHIKEVICH

The Izhikevich neuron model described above was implemented on the HNC node according to the algorithm in Listing 1. The corresponding ODE pipeline microarchitecture is shown in Fig. S3. The implemented arithmetic uses a 40-bit signed fixed-point data type (s16.23) and an explicit forward Euler method for the ODE solver with an integration step size of $h = 0.1$ ms. These design decisions were motivated by the results of an earlier study (Trensch et al., 2018) which showed that this fulfills the minimum requirements in terms of numerical precision needed to achieve sufficent simulation accuracy for the selected model.

```
FOR EACH SIMULATION TIME STEP k:
  FOR EACH NEURON n:
    -- check if ring buffer (RB) entry is valid, stage: S1 --
    IF(k_{val,n}(t_k) ≠ k) {
      i_{syn,n}(t_k) := 0
    }

    -- forward Euler, stages: S2, S3, ..., S10 --
    v_n(t_{k+1}) := v_n(t_k) + h · [ 0.04 v_n^2(t_k) + 5.0 v_n(t_k) + 140.0 − u_n(t_k) + i_n(t_k) + i_{syn,n}(t_k) + i_{ext,n}(t_k) ]
    u_n(t_{k+1}) := u_n(t_k) + h · [ ab v_n(t_k) − a u_n(t_k) ]

    -- threshold detection, stages: S11, S12 --
    IF(v_n(t_{k+1}) ≥ 30.0) {
      v_n(t_{k+1}) := c
      u_n(t_{k+1}) := u_n + d
      spikeEvent: δ(t − t_{k+1})_n
    }
```

Listing 1. Operations performed by the ODE pipeline and the implemented Izhikevich model (given as pseudo code).

The implemented operation scheduling requires 6 multipliers, 7 adders, 2 subtractors, 2 comparators, and 3 multiplexers. The pipeline requires 12 register stages indicated by the grey bars in Fig. S3. The

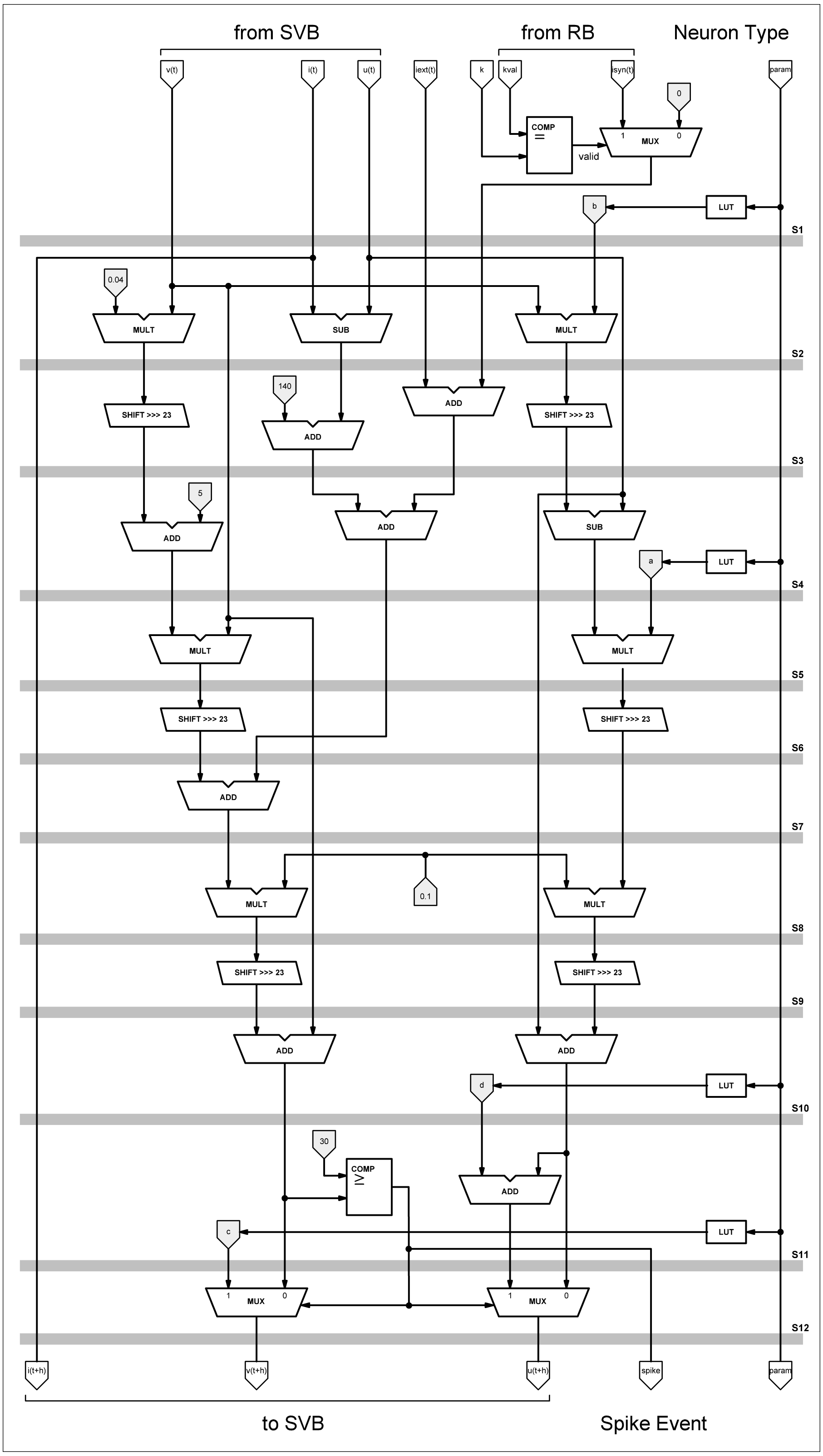

Figure S3. ODE pipeline example implementation: Izhhikevich neuron model with static synapse.

implementation of the 16 ODE pipelines requires approx. 41% of the available DSP resources, i.e., 368 DSP slices (see Table S3). Through look-up tables, the regular-spiking or fast-spiking Izhikevich neuron model type can be selected. Modeling the static synapses do not require additional operations. The value of $i(t)$ is just passed through the pipeline and may be used to apply a constant offset current.

Therefore it is also not necessary to distinguish between the accumulated excitatory and inhibitory synaptic inputs and the two can be lumped together, i.e., $i_{\text{syn}} = i_{\text{ex}} + i_{\text{inh}}$. External random input is applied through $i_{\text{ext}}(t)$ which is connected to the PRNG.

5 HARDWARE RESOURCES UTILIZATION AND POWER CONSUMPTION

Table S3 lists the utilization of the programmable logic (PL) blocks of the XCZ7045 Xilinx Zynq SoC device for the implemented HNC node prototype. The 368 DSP blocks are solely used by the ODE pipeline Izhikevich neuron model example implementation. The micro architecture of the pipeline is shown in Fig. S3. The design is limited by the available BRAM resources. Almost all BRAM blocks are utilized, which is mainly due to the current ring buffer architecture. This restricts the design in terms of the number of neurons that can be implemented and processed on a node.

The power report (Fig. S4) shows the power analysis of the implemented design. The analysis was performed using the Xilinx Vivado design tools. The total on-chip power consumption is estimated at 4.494 W. Note that the power report does not include the external memory and has a low confidence level.

Resource	Utilization	Available	Utilization %
LUT	39162	218660	17.91
LUTRAM	9367	70400	13.31
FF	50299	437200	11.50
BRAM	534	545	97.98
DSP	368	900	40.89

Table S3. Resources utilization. Utilization of the programmable logic part (PL) of the XCZ7045 Xilinx Zynq SoC device for the implemented HNC node prototype.

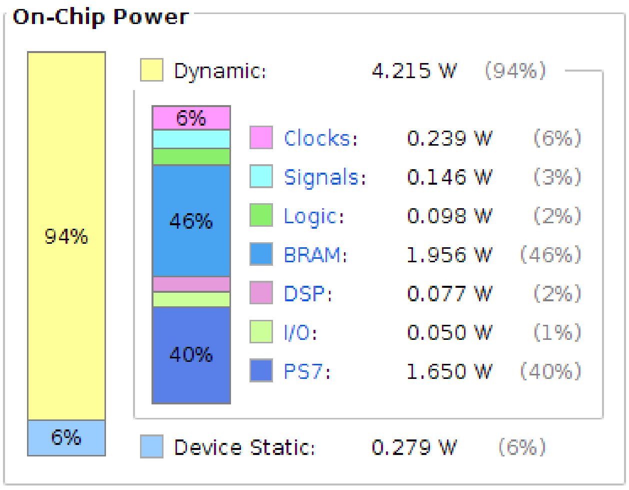

Figure S4. Power report. Xilinx Vivado on-chip power estimation for the implemented HNC node prototype design and a PL clock frequency of $f_{\text{clk}} = 200$ MHz. The total on-chip power consumption was estimated at 4.494 W.

REFERENCES

Grün, S. and Rotter, S. (eds.) (2010). *Analysis of Parallel Spike Trains* (Boston, MA: Springer US). doi:10.1007/978-1-4419-5675-0

Gutzen, R., von Papen, M., Trensch, G., Quaglio, P., Grün, S., and Denker, M. (2018). Reproducible neural network simulations: Statistical methods for model validation on the level of network activity data. *Frontiers in Neuroinformatics* 12, 90. doi:10.3389/fninf.2018.00090

Izhikevich, E. M. (2003). Simple model of spiking neurons. *Trans. Neur. Netw.* 14, 1569–1572. doi:10.1109/TNN.2003.820440

Izhikevich, E. M. (2006). Polychronization: Computation with spikes. *Neural Computation* 18, 245–282

Nordlie, E., Gewaltig, M.-O., and Plesser, H. E. (2009). Towards Reproducible Descriptions of Neuronal Network Models. *PLoS Computational Biology* 5, e1000456. doi:10.1371/journal.pcbi.1000456

Pauli, R., Weidel, P., Kunkel, S., and Morrison, A. (2018). Reproducing polychronization: A guide to maximizing the reproducibility of spiking network models. *Frontiers in Neuroinformatics* 12. doi:10.3389/fninf.2018.00046

Senk, J., Kriener, B., Djurfeldt, M., Voges, N., Jiang, H.-J., Schüttler, L., et al. (2021). Connectivity concepts in neuronal network modeling

Shinomoto, S., Shima, K., and Tanji, J. (2003). Differences in Spiking Patterns Among Cortical Neurons. *Neural Computation* 15, 2823–2842. doi:10.1162/089976603322518759

Trensch, G., Gutzen, R., Blundell, I., Denker, M., and Morrison, A. (2018). Rigorous neural network simulations: A model substantiation methodology for increasing the correctness of simulation results in the absence of experimental validation data. *Frontiers in Neuroinformatics* 12, 81. doi:10.3389/fninf.2018.00081
